# Calibration of discrete element parameters for spinach seeds and optimization of seed-metering device design

**DOI:** 10.1038/s41598-025-34245-3

**Published:** 2025-12-30

**Authors:** Jiaxing Qing, Xusheng Gong, Xuan Cai, Weijie Tan, Hanzhou Hao, Xinyi Liang, Zi’ang Gao, Chao He

**Affiliations:** 1 College of Engineering, South China Agricultural University, Guangzhou, 510642 China; 2 School of Nuclear Technology and Chemistry and Biology, Hubei University of Science and Technology, Xianning, 437100 China; 3 Hubei Key Laboratory of Radiation Chemistry and Functional Materials, Xianning, 437100 China; 4 Hubei Engineering Research Center for Fragrant Plants, Xianning, 437100 China; 5 School of Naval Architecture, Ocean and Energy Power Engineering, Wuhan University of Technology, Wuhan, 430063 China; 6 School of Artificial Intelligence and Big Data, Wuhan Business University, Wuhan, 430056 China

**Keywords:** Spinach seeds, Discrete element method (DEM), Contact parameters calibration, Seed-metering device, Qualified seeding rate, Precision planting, Engineering, Materials science

## Abstract

Due to the lack of an exact simulation model for spinach seeds, existing seed-metering devices exhibit poor seeding performance and struggle to achieve precise seeding of two seeds per seed-metering hole. This study proposes a method for calibrating discrete element parameters of spinach seeds and develops a seed-metering device with rectangular seed-metering holes tailored to the two-seeds-per-hole requirement. Firstly, a spinach seed discrete element model was constructed. Based on the Box-Behnken Design experimental scheme, simulations of angle of repose and fluidity, combined with physical of angle of repose (38.27°) and physical mass flow rate (18.60 g/s), were used to calibrate contact parameters: (1) between spinach seed models (coefficient of restitution: 0.385; coefficient of static friction: 0.481; coefficient of rolling friction: 0.042); and (2) between seed models and PVC plate (coefficient of restitution: 0.339; coefficient of static friction: 0.600; coefficient of rolling friction: 0.408). Subsequently, simulated and physical bulk density tests were conducted to verify the validity of the established spinach seed model. Guided by the agronomic requirements for spinach seed planting, the range of dimensional parameters for the seed-metering holes was defined. Using the Box-Behnken Design, simulated seeding tests were performed to optimize the device’s structural parameters (hole depth, hole length, hole width, and seed-filling angle), resulting in optimal values of 2.52 mm, 3.67 mm, 5.15 mm, and 37.99°, respectively. Finally, physical seeding tests were conducted, achieving a qualified seeding rate of 92.23% at a seeding speed of 10 r/min. These results confirm the design accuracy and high operational efficiency of the device. This study lays a foundation for the overall design of future spinach planters.

## Introduction

*Spinacia oleracea L.* (spinach) is an important leafy vegetable crop with global significance. It is mainly cultivated in China, the United States, Turkey, and Japan. Spinach is abundant in nutrients, particularly carotene, iron, and various trace elements^1^. Currently, China’s spinach cultivation area and export volume lead the world, with Chinese spinach constituting 90% of the global market^2^; However, China’s machinery for spinach cultivation is inadequate, resulting in predominantly manual planting across most regions, which leads to issues such as low operational efficiency and elevated labor intensity. Therefore, improving automation and intelligent machinery in spinach cultivation is urgently needed^2–4^.

Compared to larger seeds like corn and wheat, precision seeding of spinach faces unique challenges: its small size (equivalent particle size of 2~4 mm) increases the risk of missed seeding; its irregular spherical shape and low sphericity often cause blockage or overlap in the seeding holes; its light weight (1000-grain weight around 9 g) makes it susceptible to disturbance and drift; and it must meet the agronomic requirement of ‘two seeds per hole’. Existing seeding equipment struggles to address these issues, leading to poor seeding performance. Therefore, developing a specialized seed-metering device for spinach is crucial to improve planting efficiency and accuracy.

The essential element of spinach planting machinery is the part responsible for metering seeds, and its performance directly influences the efficacy and success rate of seeding. The research on the seed-metering device utilizes the discrete element method to set up corresponding seed models, which facilitates the simulation of the seed-metering process and thereby contributes to the structural optimization of the device^5^. DEM is a numerical method to describe the overall characteristics by simulating the kinematic and dynamic behavior of particles. In the study of precision spinach seed-metering device, DEM has significant advantages : it can simulate the interaction between particles and between particles and surface non-destructively, avoiding damage in traditional experiments; through virtual design and test, DEM can optimize seeding equipment before prototype production, improve design efficiency and accuracy; at the same time, DEM can accurately capture the complex physical interaction between seeds and equipment at the micro-scale, and deeply understand the seed behavior, so as to further optimize the equipment design and ensure accurate and efficient seeding^6–8^. Specifically, the discrete element method is applicable to simulating granular materials, enabling the analysis of their movement and fragmentation, thus offering a basis for the optimization of machinery design^9,10^. Therefore, the majority of scholars in this area of research have developed discrete element models of diverse materials and determined the parameters by integrating physical and simulated tests^11,12^. The construction and parameter calibration of discrete element models serve as the cornerstone of seed-metering device simulation. Current research predominantly employs discrete element models to simulate seed motion, with accuracy highly dependent on precise calibration of contact parameters. For example, Wei et al.^10^ calibrated the coefficient of restitution (0.400) and friction coefficient (0.450) of tobacco strips through angle of repose tests, proving that inter-particle con-tact parameters dominate simulation results. Zheng et al.^13^ proved a multi-sphere filling model for cabbage, optimizing the rolling friction coefficient to 0.040; Mi et al.^14^ combined 3D scanning to construct a sorghum seed model, calibrating the seed-material static friction coefficient to 0.345. For small-sized seeds, Wang et al.^15^ proposed that black bean parameter calibration requires controlling the kinetic energy threshold ≤ 1 × 10^−6^ J to balance computational efficiency and angle of repose accuracy. Although dynamic process simulation experiments of seed-metering devices have been conducted on various crops, there is still a lack of research related to spinach.

Seed-metering devices developed for small-sized seeds primarily fall into two categories: pneumatic and mechanical. Pneumatic seed-metering devices offer high seeding accuracy and efficiency. However, their requirement for an air supply system increases their size, making them primarily suitable for large-scale seeding equipment. This makes them clearly unsuitable for spinach seeds. In contrast, mechanical seed-metering devices, despite their relatively lower seeding accuracy and higher seed damage rate, are compact and well-suited for small seeders. Previous researchers have therefore conducted extensive work on the development of small seeders, particularly focusing on mechanical seed meters. For instance, Chen et al.^16^ set up a DEM (Discrete Element Method) model for corn seeds and reduced the multiple seeding rate to 4.83% by optimizing the spoon wheel inclination and rotational speed. Deng et al.^17^ optimized the parameters of a clip-type corn seed- metering device, achieving a field qualification rate of 90.76%. Zheng et al.^18^ designed a hyperbolic spoon-wheel seed-metering device for spinach seeds, figuring out the optimal spoon radius and quantity, with a qualification rate of 93.3%. Shang et al.^19^ developed a rice impeller seed guiding device, limiting the hole diameter to ≤ 21.7 mm and improving the hole spacing qualification rate by 6.2%. Therefore, this study aims to develop a seed-metering device suitable for small spinach seeders, focusing on mechanical seed meters. The discrete element method was employed to refine the designed seed meter, thereby reducing development costs.

To address this issue, this paper proposes a comprehensive calibration method for the contact parameters of a spinach seed discrete element model^17^. Physical experiments first figure out the characteristic parameters and the range of contact parameters for spinach seeds. A discrete element model is then developed, and simulated tests (accumulation angle and fluidity) using BBD are conducted to obtain the contact parameters between spinach seeds and PVC plates. Physical and simulated bulk density tests validate the accuracy of the parameters. Based on this model, a seed-metering device with rectangular holes is designed, considering the seed size and the agronomic requirement of double-seed filling. Simulated seed-metering tests are performed, and the optimization of seed-metering hole sizes is completed, providing a theoretical foundation for the design of precision spinach seeding equipment.

## Materials and methods

### Materials

This study takes coated spinach seeds as the research subject. The selected test instruments include an electronic vernier caliper (with an accuracy of 0.01 mm), an inclinometer (with an accuracy of 0.01°), a Phantom VEO 640 high-speed camera, a steel plate, a Polyvinyl Chloride (PVC) plate, an angle-adjustable inclined platform (hereinafter referred to as the inclined platform), a sheet of graph paper with 1-mm-sized squares in the middle (hereinafter referred to as the graph paper), a texture analyzer (with an accuracy of 0.0001 g), an electronic balance (with an accuracy of 0.01 g), a funnel (made of PVC), a beaker (made of PVC), a graduated cylinder (measurement range: 50 mL), and tweezers. All physical tests were conducted in an environment with a temperature of 26 °C and a relative humidity of approximately 50%.

### Physical property measurement of spinach seeds

#### Determination of the size of spinach seeds

**Fig. 1:**
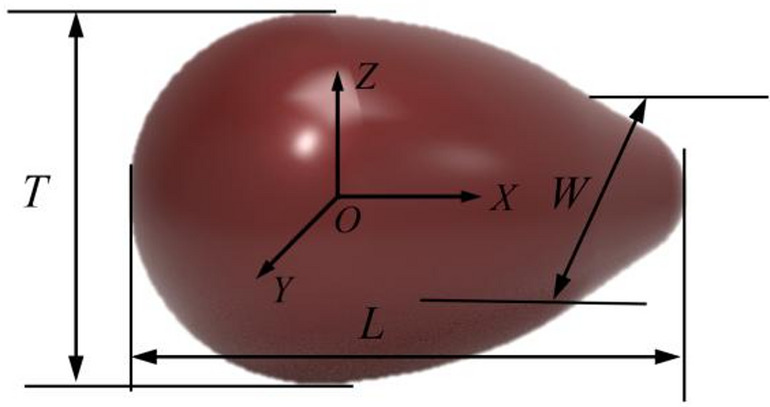
Triaxial dimensions of spinach seed.

Three hundred spinach seeds were chosen at random, and their triaxial dimensions—length (*L*), width (*W*), and thickness (*T*)—were measured using an electronic vernier caliper, as illustrated in Fig. 1. After that, the equivalent particle size (*D*_*e*_) and sphericity (*S*_*p*_) of spinach seeds were computed according to formula (1) and formula (2).

1 $${D_e}=\sqrt[3]{{LWT}}$$

where *D*_*e*_ is the equivalent particle size (mm); *L* is the length of spinach seeds (mm); *W* is the width of spinach seeds (mm); *T* is the thickness of spinach seeds (mm).

2 $${S_p}={D_e}/L \times 100\%$$

where *S*_*p*_ is the sphericity.

#### Determination of the density of spinach seeds

Ten coated spinach seeds were randomly selected as samples for density measurement. The seed density was determined using the drainage method. The procedure was as follows: a proper amount of distilled water was added to a measuring cylinder, and the initial volume of water was recorded as $\:{V}_{0}$. The mass of the 10 selected coated spinach seeds was weighed using an electronic balance, denoted as $\:{M}_{0}$. The seeds were then carefully placed in the measuring cylinder using tweezers, ensuring they were completely submerged and free of air bubbles. The total volume of the spinach seeds and distilled water in the cylinder was recorded as $\:{V}_{1}$. The density of the spinach seeds was then calculated using the formula (3). This process was repeated five times, and the average value and standard deviation were computed.

3 $$\rho =\frac{{{M_0}}}{{{V_1} - {V_0}}}$$

where $\:\rho\:$ is the density of the spinach seeds, $\:{M}_{0}$ is the mass of the spinach seeds, $\:{V}_{0}$ is the volume of distilled water before adding the spinach seeds, and $\:{V}_{1}$ is the volume of distilled water after adding the spinach seeds.

#### Determination of poisson’s ratio for spinach seeds

**Fig. 2:**
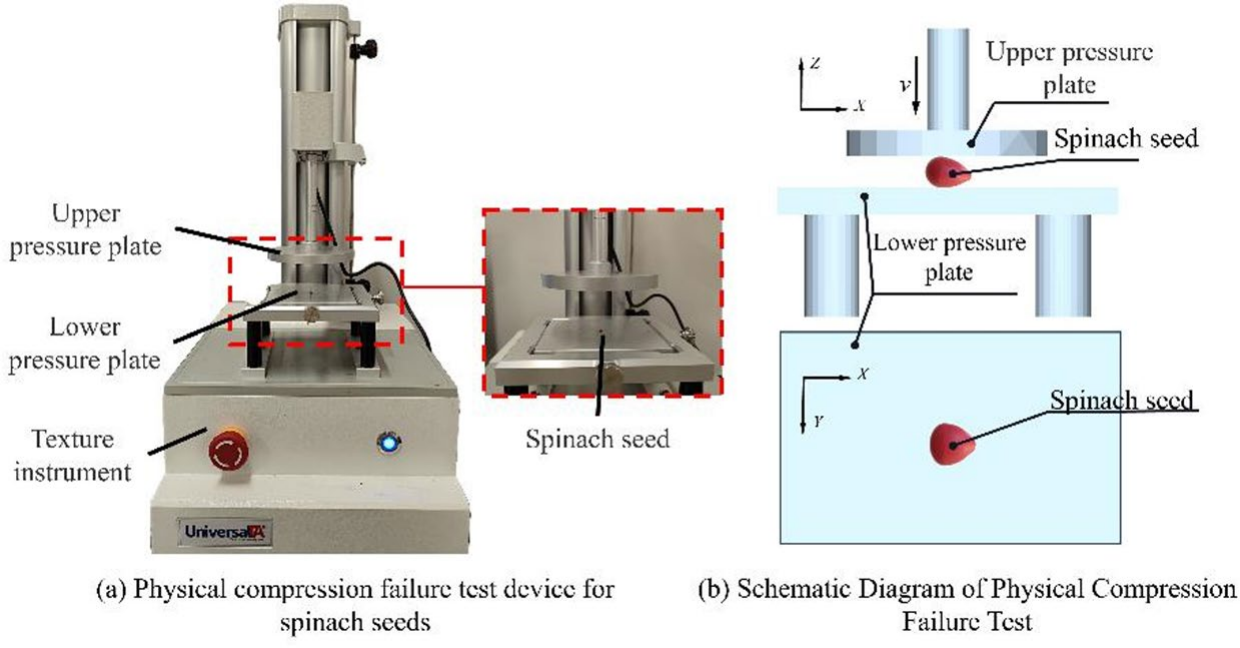
Physical compression process of spinach seeds.

A total of 14 spinach seeds were randomly selected. One seed was chosen at a time, and its three-dimensional dimensions—length (*L*), width (*W*), and thickness (*T*)—were measured using an electronic vernier caliper, as shown in Fig. 2a. The seeds were then placed on the pressure plate of a texture analyzer along the Z-direction using tweezers, as shown in Fig. 2b. The upper pressure plate of the texture analyzer applied a pre-tightening force of 1 N along the thickness direction of the spinach seeds to prevent them from moving during the compression process. Subsequently, the upper pressure plate compressed the seeds at a rate of 1 mm/s. Compression continued until the seed approached rupture, at which point the texture analyzer halted the compression and recorded the displacement (*t*) of the upper pressure plate. This displacement corresponds to the change in the seed’s thickness after compression. Subsequently, the width (*w*) of the compressed seed was measured with the electronic vernier caliper, and Poisson’s ratio of the spinach seeds was calculated using formula (4).

4 $$\mu = - \frac{{\varepsilon ^{\prime}}}{\varepsilon }=\frac{{(w - W)/W}}{{(T - t)/T}}$$

where $\mu$ is Poisson’s ratio of spinach seeds; $\varepsilon ^{\prime}$ is transverse strain of spinach seeds; $\varepsilon$ is spinach seeds axial strain; *w* is width of compressed spinach seeds (mm); *t* is the compression displacement of the upper pressure plate of the texture analyzer (mm).

#### Determination of elastic modulus of spinach seeds

The elastic modulus of small particulate materials is predominantly determined via the compression test method. In this case, twenty spinach seeds were selected. A texture analyzer was employed to compress every spinach seed along the thickness direction (Z-direction) at a speed of 1 mm/s for a displacement of 0.5 mm. After the compression process, the compression load was measured and recorded by the texture analyzer. Based on this recorded load, the elastic modulus and shear modulus of the spinach seeds can be calculated with formula (5) and formula (6).

5 $$E=\frac{{F/S}}{{\Delta T/T}}$$

where *E* is the elastic modulus of spinach seeds (MPa); *F* is the compression load when the compression displacement is 0.5 mm (N); *S* is the compression area (mm^2^); $\Delta T$is the compressive displacement of texture analyzer (mm).

6 $$G=\frac{E}{{2(1+\mu )}}$$

where *G* is the shear modulus of spinach seeds (Pa).

#### Determination of the coefficient of restitution and friction

**Fig. 3:**
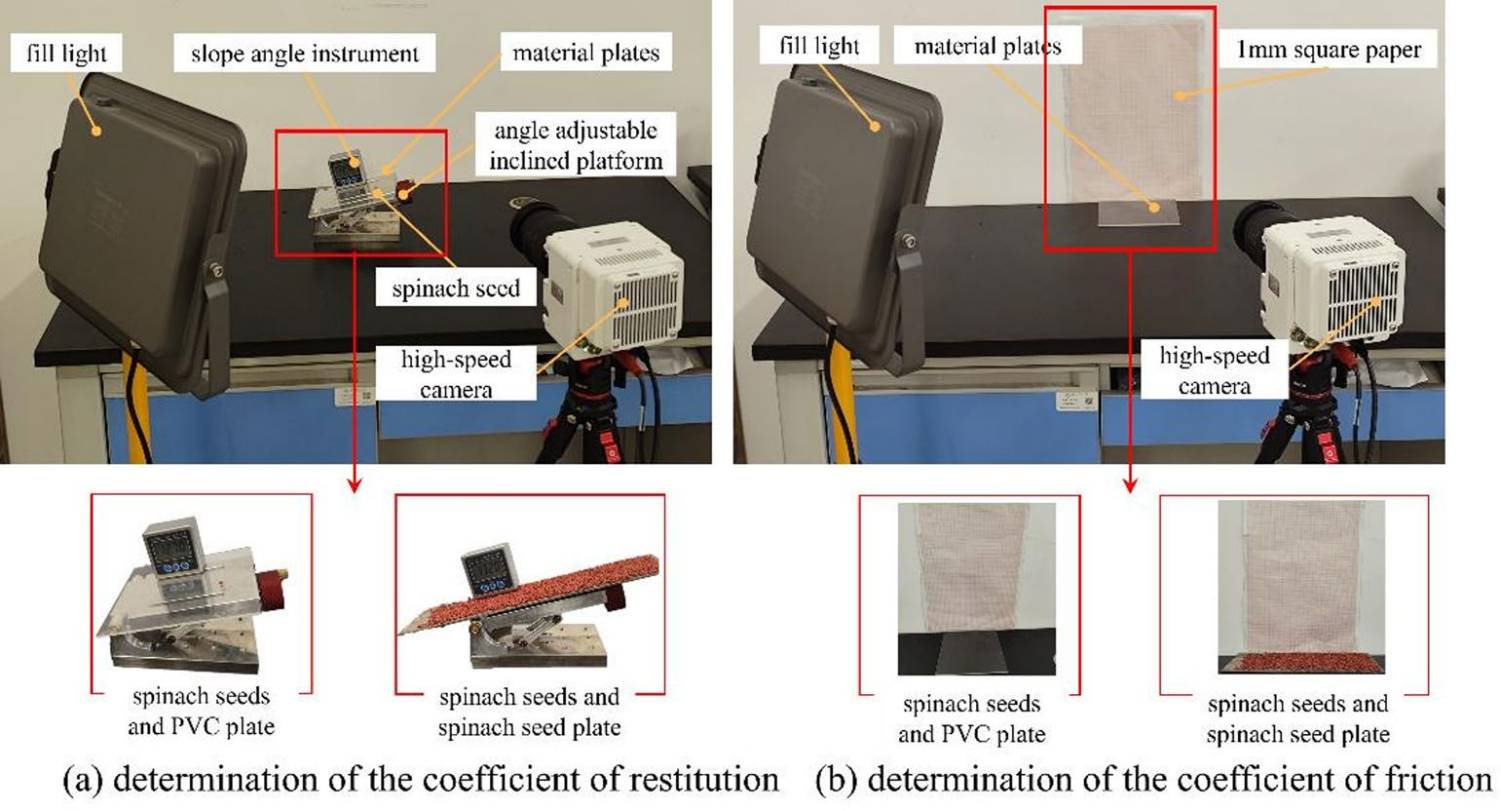
Test platform for measuring contact parameters.

During the seeding process of spinach seeds, the interaction between each component of the seed-metering device and spinach seeds is characterized by multi-point contact, involving several contact parameters: coefficient of restitution and friction coefficient^20–22^. This study constructed a contact parameter measurement test platform to ascertain the contact parameters between spinach seeds and various objects^23^, as illustrated in Fig. 3.

**Fig. 4:**
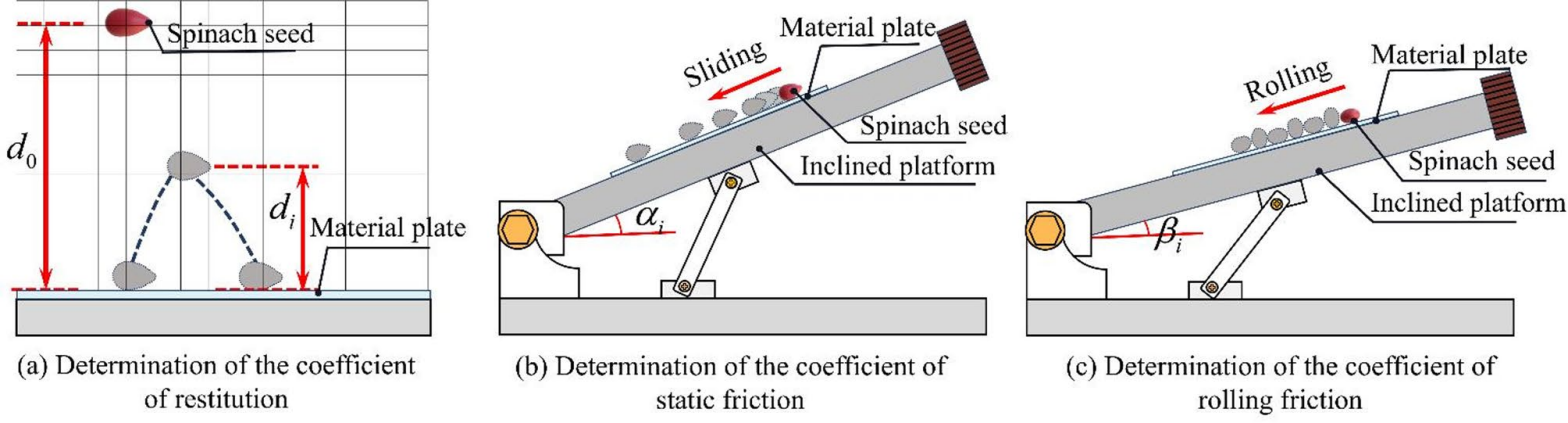
Schematic diagram of spinach seed movement during the contact parameter measurement.

This section is dedicated to measuring the coefficient of restitution for the following combinations: spinach seeds and spinach seeds, spinach seeds and PVC material, as well as spinach seeds and steel material. Figure 3a shows that the coefficient of restitution measurement platform developed in this study consists of various material plates (PVC plate and seeds plate), a high-speed camera, and a graph paper, among other components.

Fifteen spinach seeds were selected. Each seed was sequentially placed 300 mm above different material plates with tweezers and released with no initial velocity. Each spinach seed then struck the lower material plate and bounced back. The rebound behavior of every spinach seed was recorded by a high-speed camera, and the rebound heights of the seeds on different material plates are measured with the help of the graph paper^11,24^. The schematic diagram of the test process is shown in Fig. 4a. The corresponding coefficient of restitutions are calculated according to formula (7).

7 $${e_i}=\sqrt {{d_i}/{d_0}} {\text{ (}}i=1,{\text{ }}2{\mathrm{)}}$$

where *e*_1_ is the coefficient of restitution between the spinach seeds and the PVC plate; *e*_2_ is the coefficient of restitution between spinach seeds and spinach seeds; *d*_0_ is the height at which the spinach seeds were released; *d*_1_ is the height at which the spinach seeds rebounded after colliding with the PVC plate (mm); *d*_2_ is the height at which the spinach seeds rebounded after colliding with the seed plate (mm).

The friction coefficient is categorized into coefficients of static and rolling friction^25,26^. This section will measure the friction coefficient between various objects: spinach seeds and PVC material, spinach seeds and spinach seeds. Figure 3b illustrates that the friction coefficient measurement apparatus forms an inclined platform, various material plates (PVC plate, seeds plate), a high-speed camera, and an inclinometer and other components.

In this study, fifteen spinach seeds were randomly selected, and each seed was placed on a PVC plate or a seed plate respectively—both plates were placed on the inclined platform. The angle of the inclined platform was gradually adjusted until each spinach seed started to slide; at this moment, a high-speed camera captured the image of the seed, and the angle value was recorded using the inclinometer. The schematic diagram of the test process is shown on Fig. 4b. The coefficient of static friction between spinach seeds and the various materials (PVC plate and seed plate) was calculated according to formula (8).

8 $${f_i}=\tan {\alpha _i}{\text{ }}(i=1,2)$$

where *f*_1_ is the coefficient of static friction between spinach seeds and PVC plate; *f*_2_ is the coefficient of static friction between spinach seeds and spinach seeds; $\:{\alpha\:}_{1}$ is the tilt angle of the inclined platform when the spinach seed began to slide on the PVC plate (°); $\:{\alpha\:}_{2}$ is the tilt angle of the inclined platform when the spinach seed began to slide the steel plate (°).

Fifteen spinach seeds with higher sphericity were randomly selected in this experiment. Each seed was then placed on the PVC plate, which was placed on the inclined platform. Next, the angle of inclination of the inclined platform was gradually adjusted. As soon as the spinach seed started to roll, the high-speed camera captured this moment and simultaneously recorded the angle shown on the inclinometer of the inclined platform. The schematic diagram of the test process is shown in Fig. 4c. Subsequently, the coefficient of rolling friction between spinach seeds and the PVC plate can be calculated according to formula (9).

9 $${r_1}=\tan {\beta _1}$$

where *r*_1_ is the coefficient of rolling friction between spinach seeds and PVC plate; $\:{\beta\:}_{1}$ is the tilt angle of the inclined platform when the spinach seed rolls on the PVC material plate (°).

### Establishment of discrete element model

This paper established the discrete element model of spinach seeds (hereinafter referred to as the spinach seed DEM model) and adopted the Hertz-Mindlin (no slip) model as the contact model between the simulated particles. Subsequently, we conducted a simulated angle of repose for the model to get the parameters of the contact model^29,30^.

**Fig. 5:**
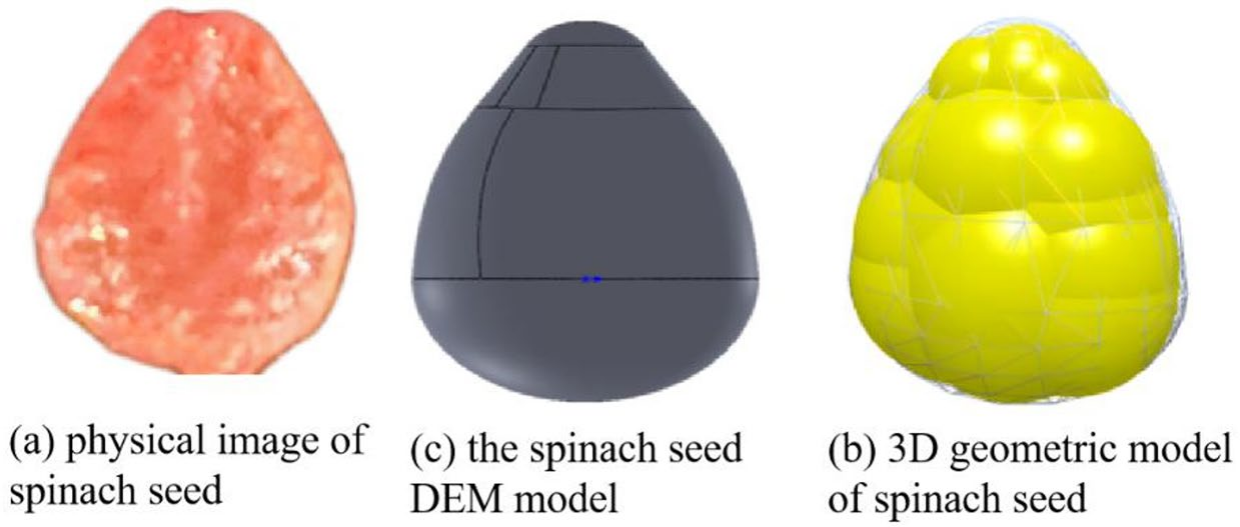
Spinach seed modeling.

The following is the process of establishing the spinach seed DEM model in this study. Initially, based on the average dimensions of spinach seeds, a three-dimensional model of the seeds was created using SOLIDWORKS 2019 software. Then, the constructed three-dimensional model was imported into EDEM 2022 software. In this software, it was represented by 21 spheres to create a spinach seed DEM model, as illustrated in Fig. 5.

### Calibration of contact parameters between spinach seeds

#### Physical angle of repose test

**Fig. 6:**
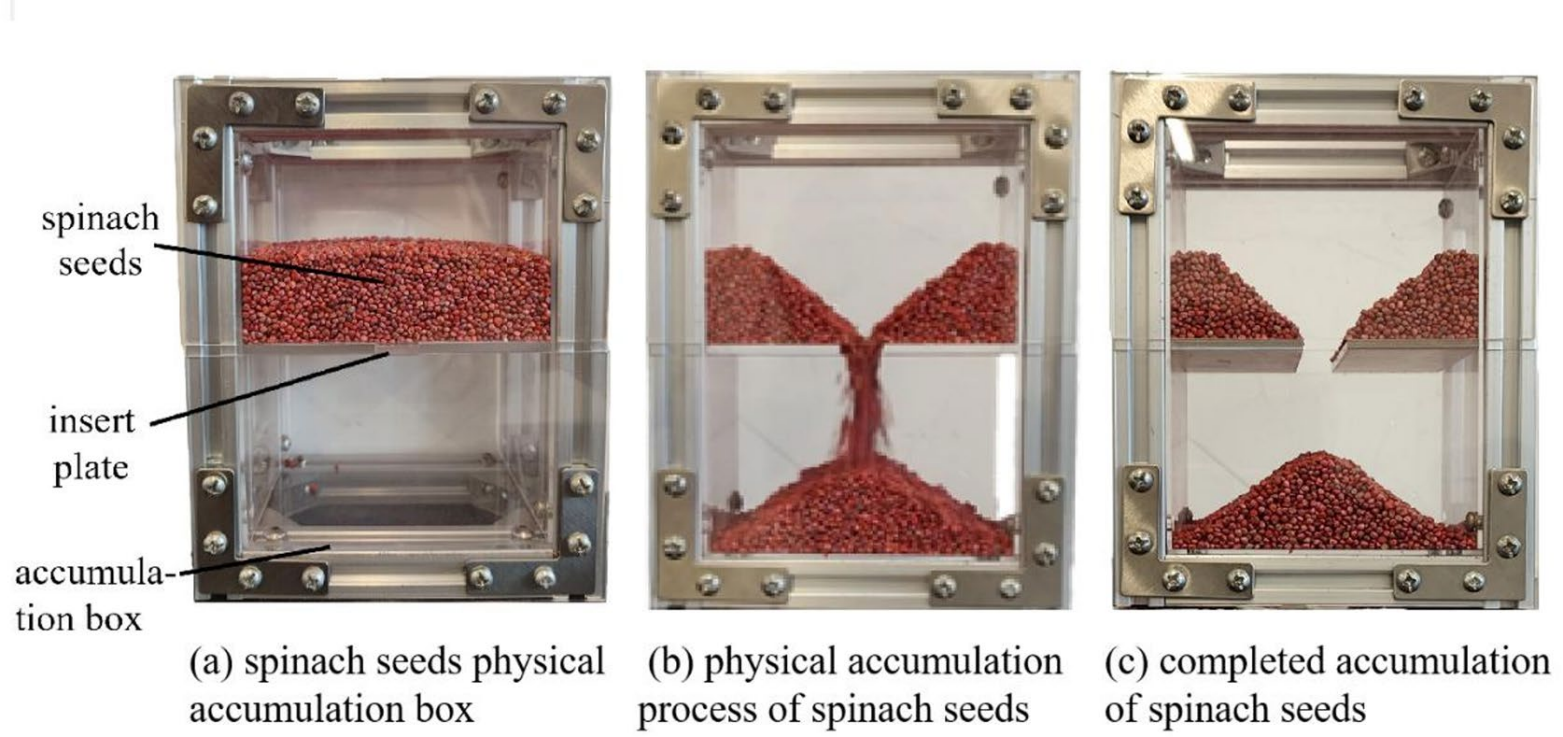
Process of physical angle of repose test.

The angle of repose of granular materials can indicate the frictional properties and characteristics of collision among particles^13,27^. The angle of repose test is frequently employed in many studies to ascertain the contact parameters of materials. This paper utilizes a self-designed accumulation box to conduct physical angle of repose tests on spinach seeds, subsequently calibrating the contact parameters of the seeds. The testing procedure is illustrated in Fig. 6.

At the start of the experiment, 300 g of (approximately 20,000 seeds) spinach seeds were deposited in the upper section of the accumulation box (the internal material is made of PVC), then gently agitated to level the seed surface, and then the insert plate was removed. The spinach seeds dispersed independently and gradually accumulated. When the spinach seeds ceased to flow and remained approximately stationary, the camera captured an image of their physical angle of repose.

**Fig. 7:**
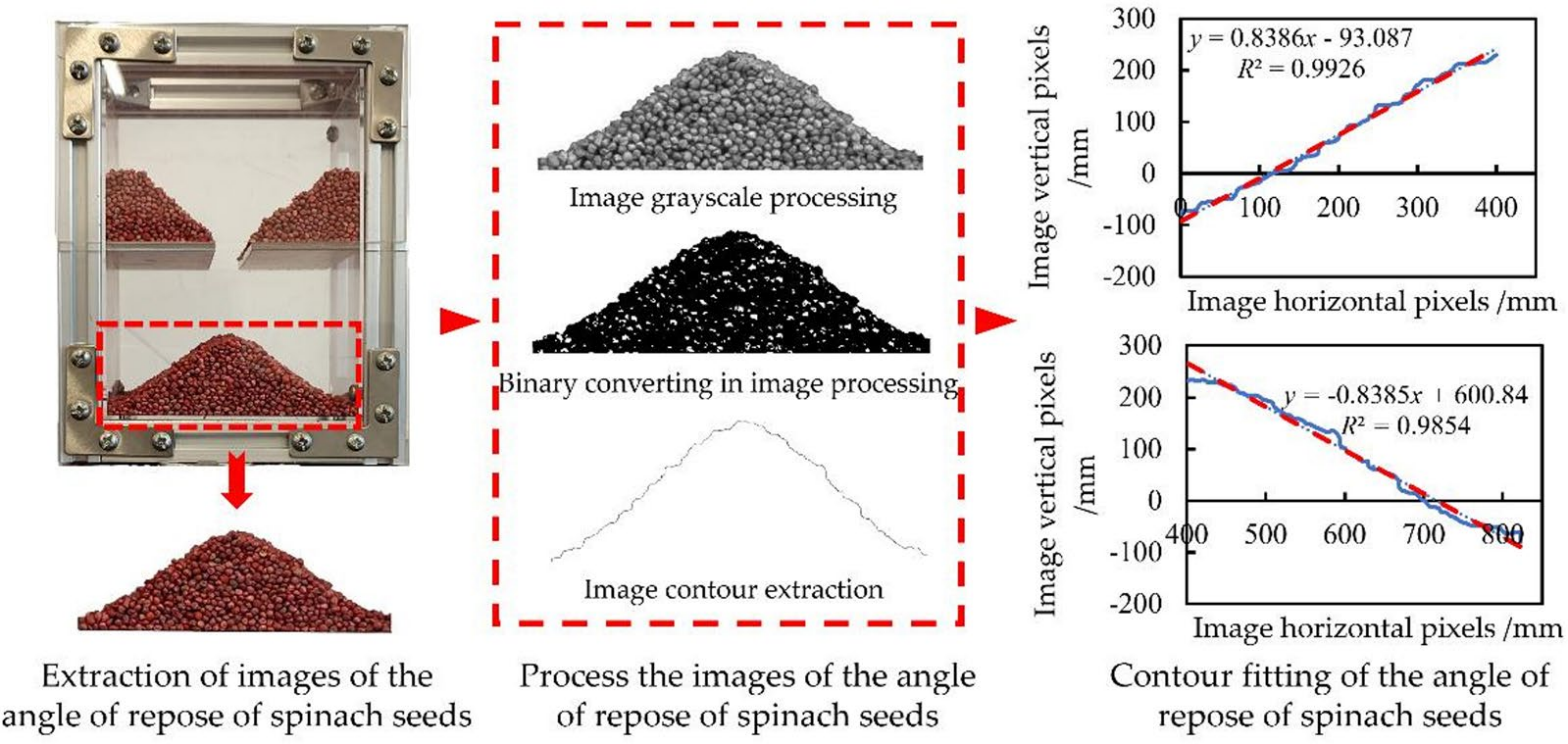
Image processing process of physical angle of repose.

To obtain the results of the physical angle of repose test of spinach seeds, the collected images must undergo processing^28^, as illustrated in Fig. 7. Initially, Adobe Photoshop 2021 (v22.0) software was used to isolate and eliminate the background of the physical accumulation image individually. The processed image was subsequently imported into MATLAB R2024a, a software utilized for completing the grayscale conversion and binarization of the image, followed by the extraction of the image’s contour pixels. Ultimately, Microsoft Excel was employed to perform a linear fit on the contour pixels, thereby determining the slope of the contour pixels on either side of the image of angle of repose, which facilitated the calculation of the physical angle of repose (*R*) of spinach seeds. Perform the physical angle of repose test ten times and calculate the mean and standard deviation.

#### Simulated angle of repose test

**Fig. 8:**
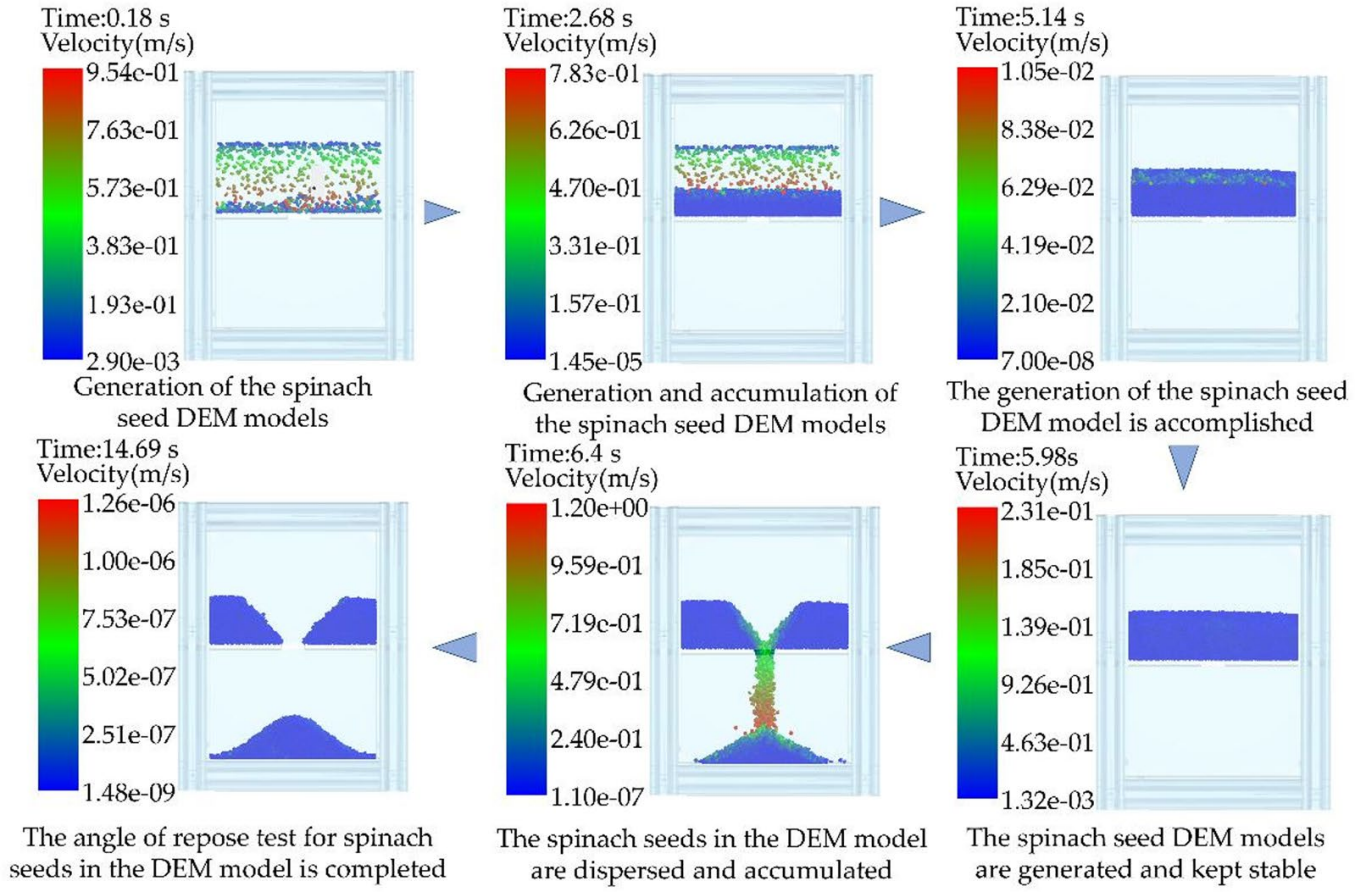
The procedure for conducting the simulated angle of repose test.

The three-dimensional model of the accumulation box was imported into the EDEM 2022 software. Additionally, 20,000 spinach seed DEM models were generated on the upper layer of the accumulation box model. After the spinach seed DEM models stabilized, the insert plate in the accumulation box model was instantaneously removed. The models dispersed to create a simulated angle of repose, resulting in the acquisition of the image of simulated angle of repose. Then, the processing flow identical to that of the image of physical angle of repose test was completed, resulting in the acquisition of the simulated angle of repose of the models. The process of the simulated angle of repose test is illustrated in Fig. 8.

#### Plackett-Burman test

Given the multiplicity of contact parameters in spinach seed modeling, a screening experiment was implemented to identify statistically significant factors affecting simulated repose angle. Table 1, a method to calibrate discrete element parameters presents the key contact parameters for the spinach seed DEM model. Using previously measured contact parameters as experimental factors and the simulated repose angle as the response variable, a Plackett-Burman experimental design was executed to determine influential parameters. The corresponding experimental levels are detailed in Table 2.

**Table 1:** Results of contact parameters of spinach seeds.

No.	Test object	Test parameter	Value
1	Spinach seeds and PVC plate	Coefficient of restitution	0.30 ~ 0.65
		Coefficient of static friction	0.30 ~ 0.62
		Coefficient of rolling friction	0.15 ~ 0.45
2	Spinach seeds and spinach seeds	Coefficient of restitution	0.20 ~ 0.50
		Coefficient of static friction	0.30 ~ 0.60
		Coefficient of rolling friction	0.01 ~ 0.09^15^

**Table 2:** Experimental parameters and levels of the Plackett-Burman test.

Experimental parameter	Level		
	Low (− 1)	Middle (0)	High (+ 1)
Coefficient of restitution between spinach seeds and spinach seeds (*X*_1_)	0.20	0.3	0.50
Coefficient of static friction between spinach seeds and spinach seeds (*X*_2_)	0.30	0.45	0.60
Coefficient of rolling friction between spinach seeds and spinach seeds (*X*_3_)	0.01	0.05	0.09
Coefficient of restitution between the spinach seeds and the PVC plate (*X*_4_)	0.30	0.475	0.65
Coefficient of static friction between spinach seeds and PVC plate (*X*_5_)	0.30	0.60	0.62
Coefficient of rolling friction between spinach seeds and PVC plate (*X*_6_)	0.15	0.30	0.45

#### Box-Behnken design test

The results of the Plackett-Burman test wrote down those three experimental parameters (*X*_1_, *X*_2_, and *X*_3_) had a major influence on the simulated angle of repose. To further identify the key contact parameters between the spinach seed DEM models, three contact parameters related to spinach seeds were selected as test factors, with the simulated angle of repose from the spinach seed DEM models used as the response variable. The experiment was conducted following the Box-Behnken design methodology. The experimental parameters and their corresponding levels are presented in Table 3.

**Table 3:** Experimental parameters and levels of the Box-Behnken design test.

Experimental parameter	Level		
	Low (− 1)	Middle (0)	High (+ 1)
*X*_1_	0.20	0.30	0.50
*X*_2_	0.30	0.45	0.60
*X*_3_	0.01	0.05	0.09

### Calibration of spinach seed-contact material contact parameters

#### Physical fluidity test

Following the calibration of inter-seed contact parameters via physical and simulated angle of repose tests, the contact parameters between spinach seeds and the PVC plate were further calibrated using a fluidity test. The flow characteristics of the spinach seeds were evaluated based on mass flow rate.

**Fig. 9:**
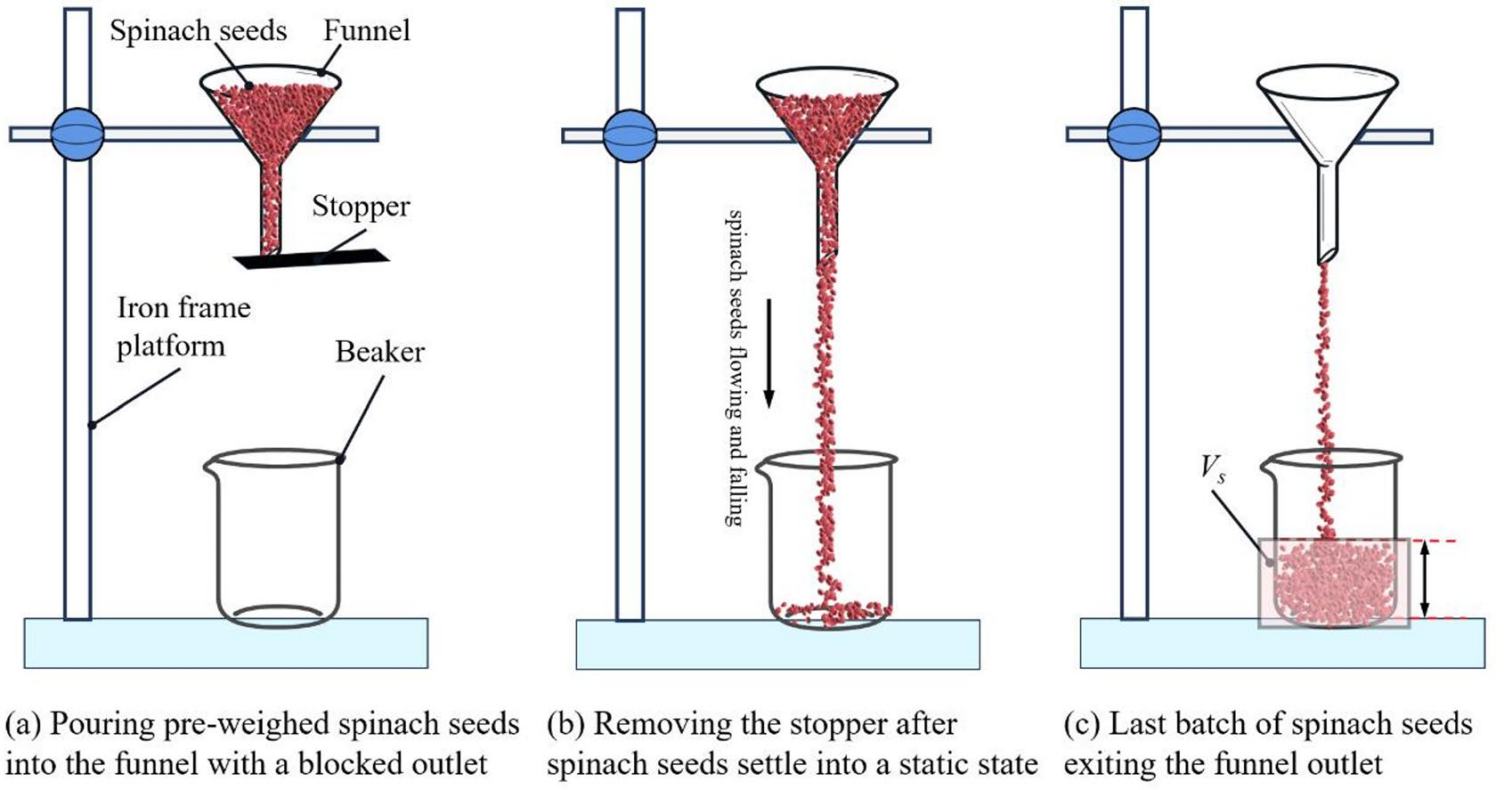
Schematic of the physical fluidity test procedure.

For the physical fluidity test, a batch of pre-weighed spinach seeds (*m*_*s*_ = 60.02 g) was used. The funnel was mounted on an iron frame platform above the center of the beaker, with its outlet positioned 100 mm vertically above the beaker’s rim. The funnel outlet was blocked with a stopper, illustrated in Fig. 9a. The pre-weighed seeds were poured into the funnel, and after the seeds reached a static state, the stopper was removed to allow the seeds to flow freely through the funnel, illustrated in Fig. 9b and c. The entire process—from the onset of flow to the discharge of the final seed—was recorded using a high-speed camera. The time instances when the first and last seeds exited the funnel outlet were defined as *t*_1_ and *t*_2_, respectively. The physical mass flow rate was then calculated using formula (10). The test was repeated ten times, and the average mass flow rate along with its standard deviation was calculated.

10 $${V_f}=\frac{{{m_s}}}{{{t_2} - {t_1}}}$$

where *V*_*f*_ is the physical mass flow rate of spinach seeds (g/s); *m*_*s*_ is the mass of spinach seeds (g); *t*_1_ is the time when the first seed leaves the funnel outlet (s); *t*_2_ is the time when the last seed exits the funnel outlet (s).

#### Simulated fluidity test

To determine the simulated mass flow rate of the spinach seed DEM models, a 3D model of the funnel, iron frame platform, and receiving beaker was constructed using SOLIDWORKS 2019 at a 1:1 scale according to their actual dimensions. The funnel model was fixed directly above the receiving beaker model via the iron frame platform model, with the vertical distance between the funnel’s outlet and the beaker’s rim set to 100 mm.

**Fig. 10:**
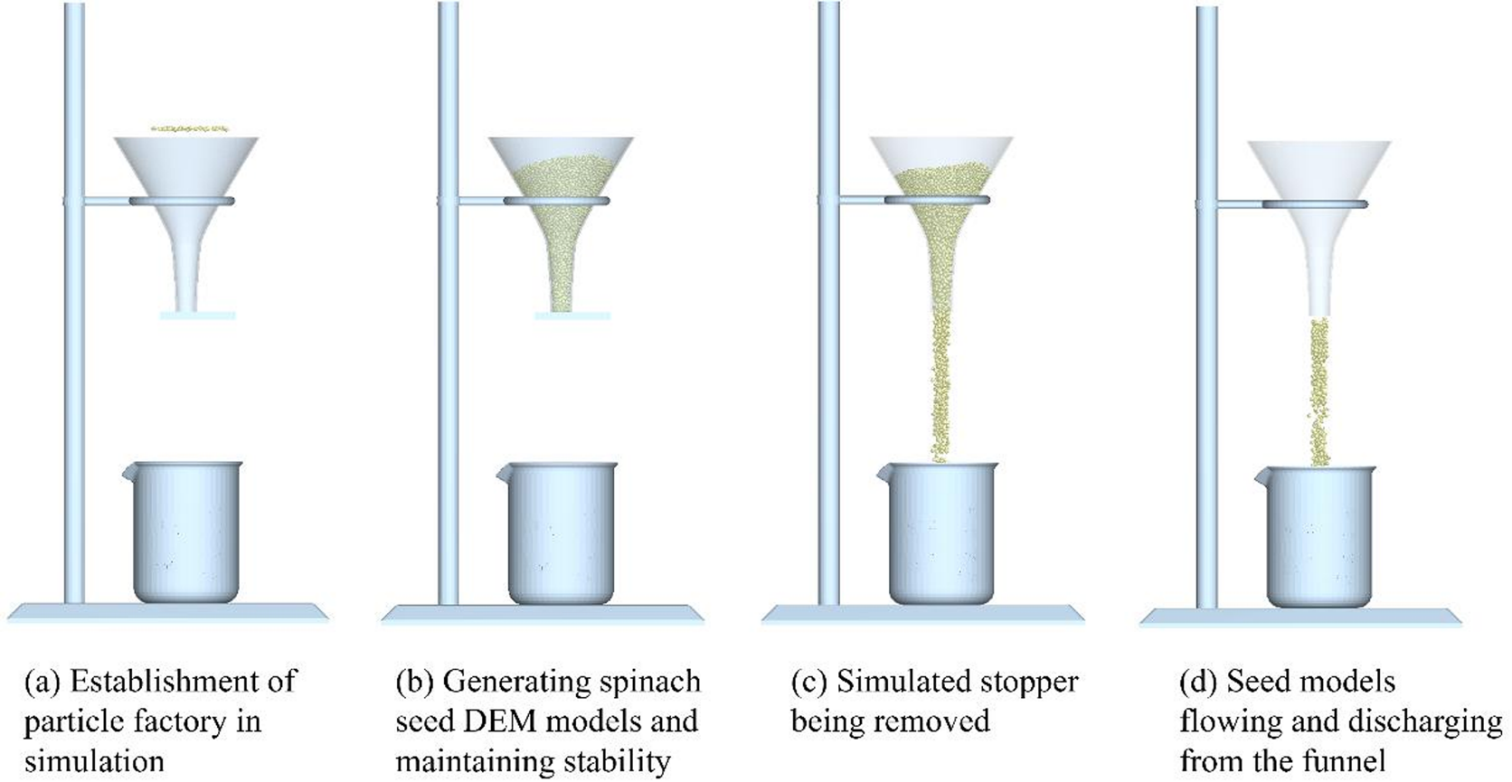
Schematic of the simulated fluidity test procedure.

Subsequently, this 3D assembly model was imported into EDEM 2022. A simulated stopper (dimensions: 50 mm × 50 mm) was placed at the funnel’s bottom outlet, and a particle factory (dimensions: 50 mm × 50 mm) was established at the center of the funnel’s top, illustrated in Fig. 10a. The particle factory generated spinach seed DEM models with a total mass of 60.02 g, which were directed vertically toward the funnel plane at a speed of 0.01 m/s—resulting in the accumulation of seed models within the funnel, illustrated in Fig. 10b. Once the spinach seed DEM models ceased motion and their speed approached zero, the simulated stopper was removed, allowing the seed models to flow and discharge from the funnel, illustrated in Fig. 10c,d.

The time when the first seed model exited the funnel outlet of the simulated funnel was recorded as *t*_*s*1_, and the time when the last seed model exited was recorded as *t*_*s*2_. The simulated mass flow rate was then calculated using formula (11):

11 $${V_{fs}}=\frac{{{m_s}_{s}}}{{{t_{s2}} - {t_{s1}}}}$$

where *V*_*fs*_ is the simulated mass flow rate (g/s); *m*_*ss*_ is the mass of the spinach seed DEM model (g); *t*_*s*1_ is the time when the first spinach seed DEM model exits the outlet of the simulated funnel (s); *t*_*s*2_ is the time when the last spinach seed DEM model exits the outlet of the simulated funnel (s).

#### Box-Behnken design test

Based on the comparison between the physical and simulated angles of repose of spinach seeds, it was demonstrated that the contact parameters between the spinach seed DEM models and contact materials have no significant effect on the simulated angle of repose. Therefore, a simulated fluidity test was conducted in this study to calibrate the contact parameters between spinach seeds and contact materials (specifically PVC). Three contact parameters between spinach seeds and PVC were selected as test variables, and their levels were set according to the previously determined value ranges (Table 4). With the simulated mass flow rate of the spinach seed DEM models as the response variable, the BBD was applied to perform the simulated fluidity test.

**Table 4:** Experimental factors and levels of the BBD for contact parameters between spinach seeds and PVC plate.

Experimental parameter	Level		
	Low (− 1)	Middle (0)	High (+ 1)
*X*_4_	0.30	0.475	0.65
*X*_5_	0.30	0.46	0.62
*X*_6_	0.15	0.30	0.45

### Parameter validation of the spinach seed DEM model

Following the completion of physical and simulated angle of repose tests as well as fluidity tests, the contact parameters between spinach seed DEM models and between these models and contact materials were finalized. To verify the accuracy of these calibrated parameters and confirm the rationality of the established spinach seed DEM models, further physical and simulated bulk density tests were conducted.

#### Physical bulk density test

**Fig. 11:**
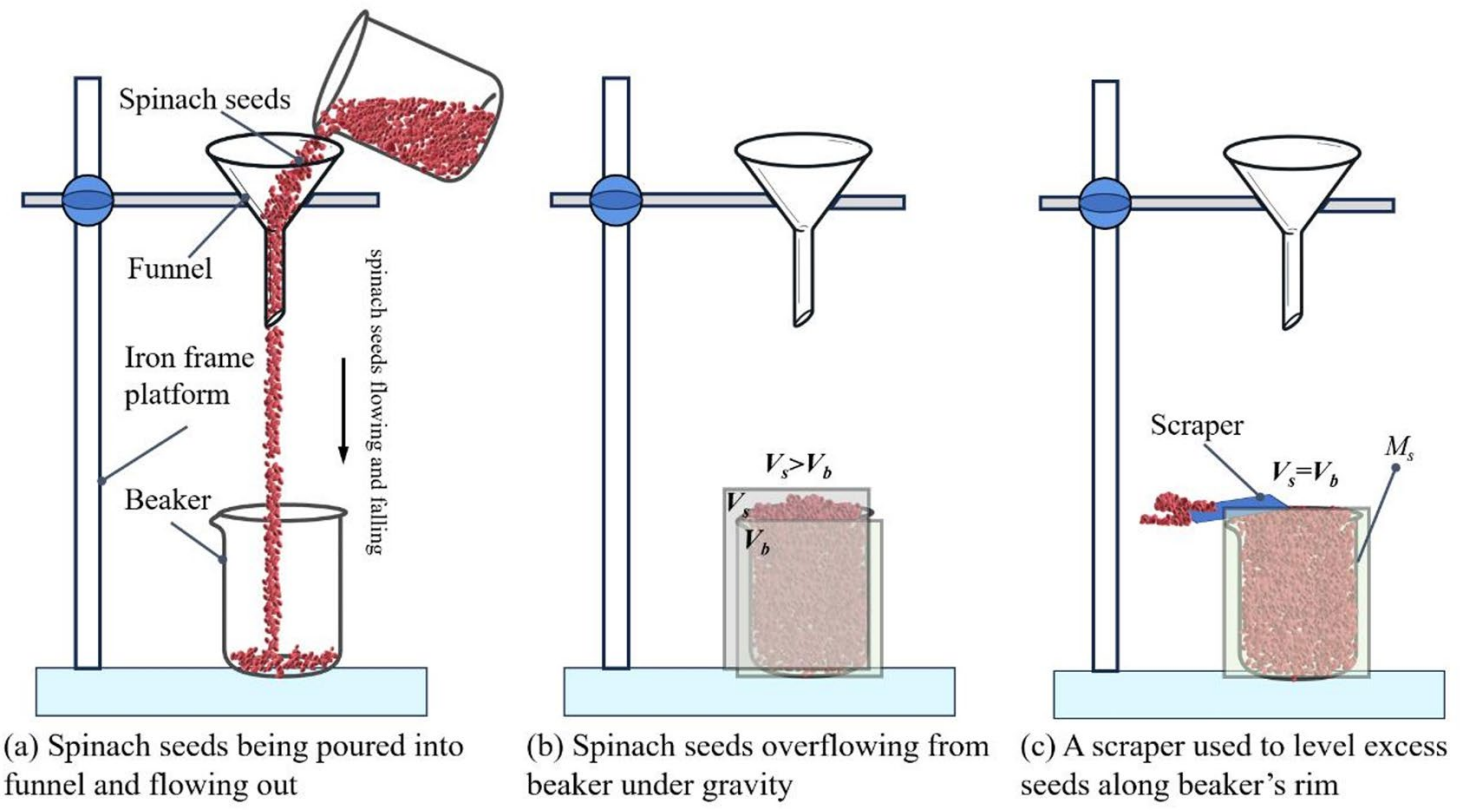
Schematic of the physical bulk density test procedure.

To determine the physical bulk density ($\:{\rho\:}_{b}$) of spinach seeds in a free-filling state, the experiment utilized a graduated beaker (nominal capacity: 200 mL; measured full volume: *V*_*b*_ = 256.42 mL), an electronic balance, an iron frame platform, a funnel, and a scraper. The test procedure was as follows:

The funnel was fixed above the beaker via the iron frame platform, with the vertical distance between the funnel outlet and the beaker’s rim set to 50 mm, 100 mm, and 150 mm—resulting in three test groups. Spinach seeds were slowly and continuously poured into the funnel at a rate of approximately 0.01 m/s, allowing them to fill the beaker naturally under gravity until overflow was observed, illustrated in Fig. 11a,b. At this point, the filling volume (*V*_*s*_) exceeded the beaker’s full volume (*V*_*s*_> *V*_*b*_).

Next, a scraper was used to level the excess seeds along the beaker’s rim, with the scraped seeds discarded to prevent them from falling back into the beaker. During this process, the beaker was not vibrated or compacted, resulting in a final filling volume equal to the beaker’s full volume (*V*_*s*_ = *V*_*b*_), illustrated in Fig. 11c. The total mass of the beaker plus the leveled seeds (*M*_*t*_) was then weighed, along with the mass of the empty beaker (*M*_*b*_).

The physical bulk density of the spinach seeds was calculated using formula (12). Each test group was repeated five times, and the mean and standard deviation of the physical bulk density were calculated for each group.

12 $${\rho _b}=\frac{{{M_s}}}{{{V_b}}}=\frac{{{M_t} - {M_b}}}{{{V_b}}}$$

where $\:{\rho\:}_{b}$ is the physical bulk density of spinach seeds (g/cm^3^), *M*_*s*_ is the mass of the leveled seeds (g), *M*_*t*_ is the total mass of the beaker and the leveled seeds (g), *M*_*b*_ is the mass of the empty beaker (g), *V*_*b*_ is the measured full volume of the beaker ($\:{\mathrm{cm}}^{3}$).

#### Simulated bulk density test

To determine the simulated bulk density ($\:{\rho\:}_{bs}$) of the spinach seed DEM models, a 3D model of the funnel, iron frame platform, and beaker was constructed in SOLIDWORKS 2019 based on their actual dimensions. The funnel model was positioned directly above the graduated beaker model via the iron frame platform, with the vertical distance between the funnel’s lower port and the beaker’s rim to 100 mm, 150 mm, and 200 mm—corresponding to three separate test groups.

**Fig. 12:**
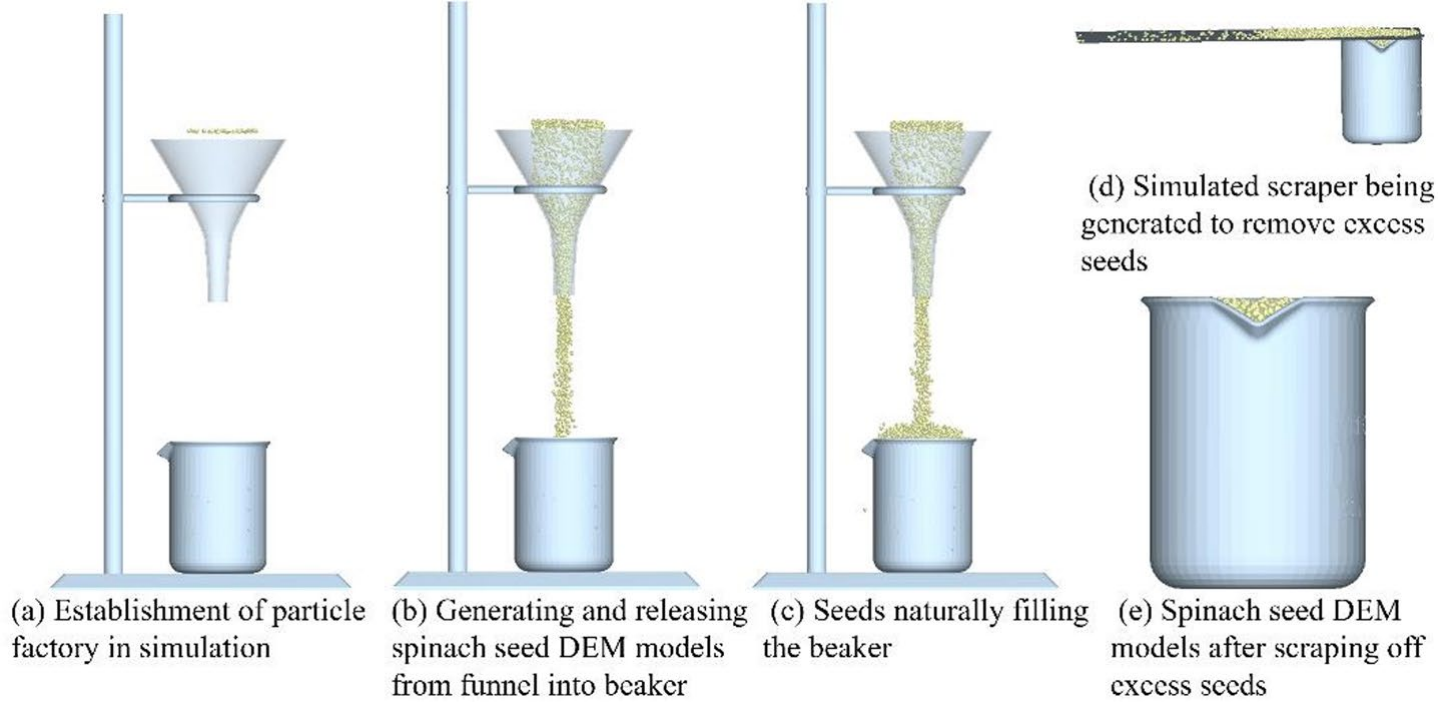
Schematic of the simulated bulk density test procedure.

This 3D model was then imported into EDEM 2022. To ensure consistency with the physical bulk density accumulation test, a 50 mm × 50 mm particle factory was created at the top of the simulated funnel, illustrated in Fig. 12a. Positioned perpendicular to the funnel’s inner plane, the particle factory generated spinach seed DEM models at a speed of 0.01 m/s, which were released from the funnel into the beaker, illustrated in Fig. 12b. Once the seeds naturally filled the beaker and reached a stable state, a simulated scraper was generated to remove excess seeds above the rim of the simulated beaker and move them out of the simulation area, illustrated in Fig. 12c, d and e.

The total mass (*M*_*ss*_) of the spinach seed DEM models in the beaker was obtained via the Solve Report interface of EDEM 2022. The simulated bulk density ($\:{\rho\:}_{bs}$) of the spinach seed DEM models was then calculated using formula (13):

13 $${\rho _b}_{s}=\frac{{{M_{ss}}}}{{{V_{bs}}}}$$

where $\:{\rho\:}_{bs}$ is the simulated bulk density of the spinach seed DEM model (g/cm^3^), and *M*_*ss*_ is the total mass of the spinach seed DEM models after excess seeds were scraped off (g); *V*_*bs*_ is the full volume of the simulated beaker (cm^3^).

### Optimization and design of spinach seed-metering device

#### Structural design and performance validation

**Fig. 13:**
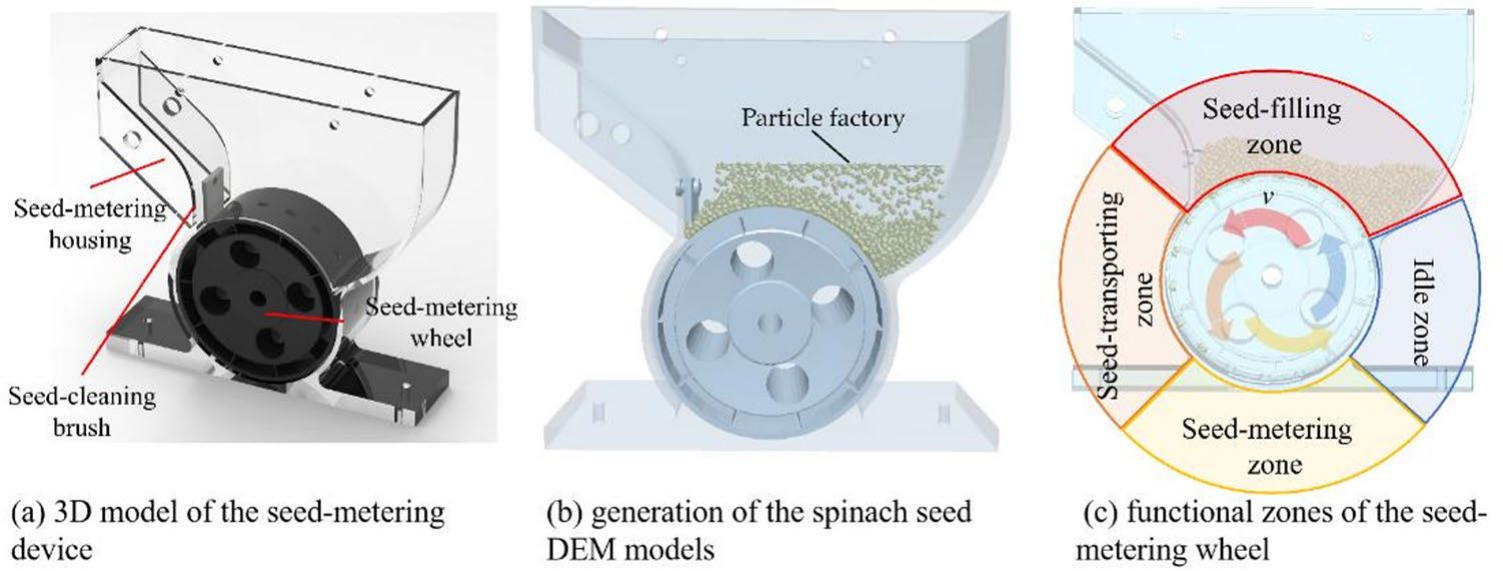
Design of the spinach seed-metering device.

The spinach seed-metering device consists of a seed-metering housing, a seed-cleaning brush, and a seed-metering wheel, with the latter serving as the core functional component holding seed-metering holes (Fig. 13a). During operation, seeds enter these holes as the wheel rotates uniformly. Functionally, the rotational cycle is divided into four sequential zones: seed-filling, seed-transporting, seed-metering, and idle zones. In the seed-filling zone, seeds settle into the holes driven by gravity and collisions between seeds. Upon entering the seed-transporting zone, the wheel transports the seeds contained within the holes. In the seed-metering zone, the seeds are released to complete the seeding process. Finally, in the idle zone, emptied holes are reset before returning to the seed-filling zone, enabling continuous spinach seeding. To improve the germination rate of spinach seeds and in line with the agronomic requirements for direct spinach seeding, two seeds should be discharged per planting hole during the seeding process.

**Fig. 14:**
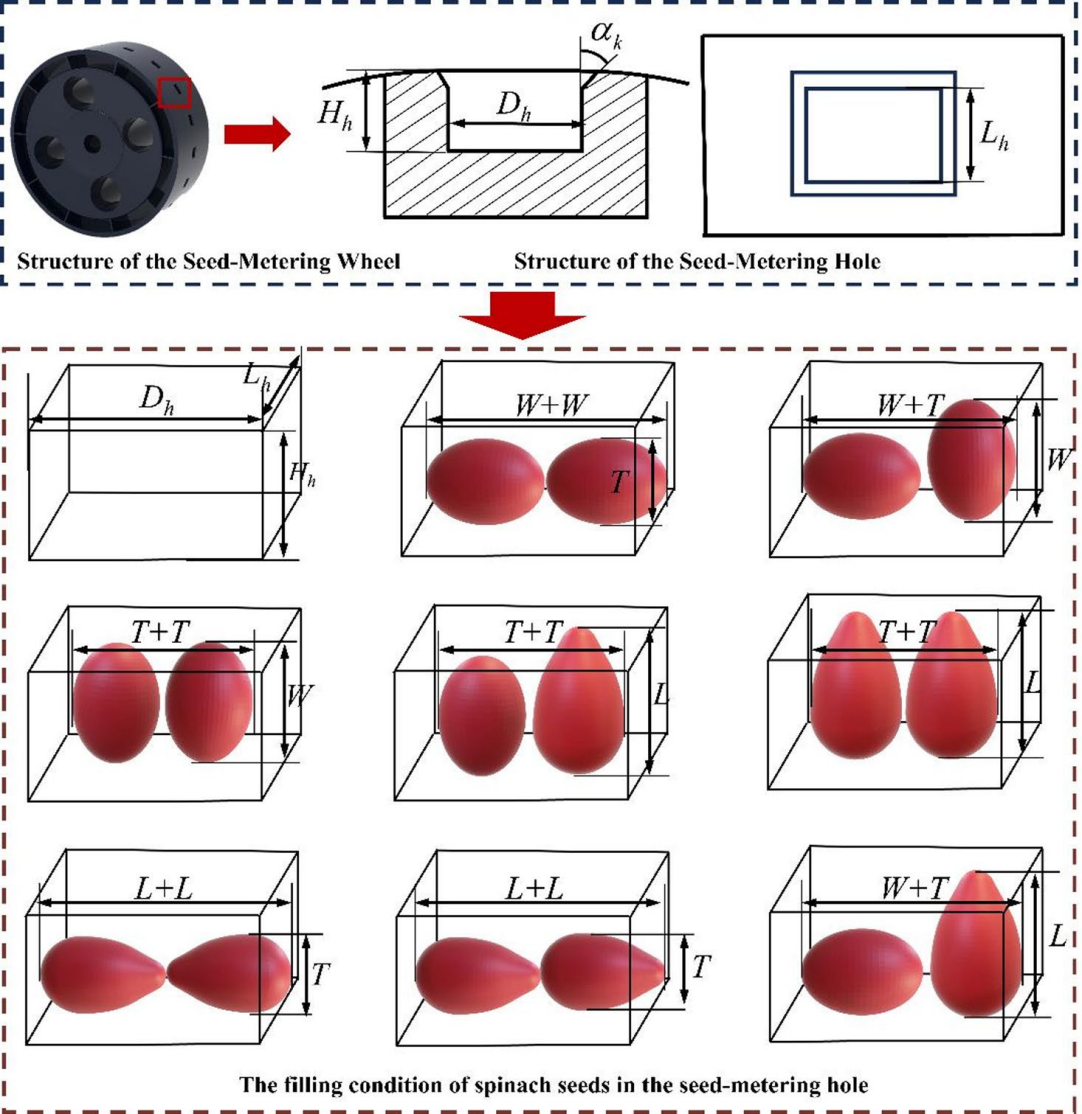
Parametric configuration of the seed-metering wheel and seed-filling dynamics within the seed-metering holes.

The dimensional parameters of the seed-metering holes (*H*_*h*_, *L*_*h*_, *D*_*h*_), which govern seed-filling performance, constitute the functional core of the Seed-metering wheel. Three-dimensional morphological analysis revealed significant asphericity in spinach seeds, prompting optimization of a rectangular hole configuration. Figure 14 parametrically defines the hole height (*H*_*h*_), length (*L*_*h*_), and width (*D*_*h*_).

Based on the seeding requirements for spinach seeds, the dimensions of the seed-metering holes in the seed-metering wheel were initially designed as follows: the depth of the seed-metering hole is 3 mm, the length is 4.5 mm, and the width is 5.5 mm. Using these preliminary dimensions, a 3D model of the seed-metering device was created. The 3D model of the designed seed-metering device was then imported into EDEM 2022 for simulation. Within EDEM, a particle factory positioned 20 mm above the device directly generated 5000 spinach seed DEM models (Fig. 13b). With the seed-metering wheel operating at 10 r/min, the simulated parameters were set as follows: time step = 4.28068 × 10^− 8^ s and data saving interval = 0.01s. After running the simulation, the functional zones of the seed-metering wheel were visualized (Fig. 13c). Finally, particle trajectories exported through EDEM’s post-processor were used to simulate and validate the overall seeding performance of the device, ultimately yielding the simulated qualified seeding rate (calculated using formula (14)) for the preliminary design of the seed-metering device. The simulated seeding test was repeated five times, and the mean value and standard deviation of the simulated qualified seeding rate were calculated.

14 $${Q_s}={Z_1}/Z \times 100\%$$

where *Q*_*S*_ is the simulated qualified seeding rate; *Z*_*1*_ is the number of seed-metering holes with 2 seeding during the simulated seeding process; *Z* is the total number of seed-metering holes during the simulated seeding process.

#### Seed-metering wheel optimization design

Agronomic requirements for spinach cultivation mandate biphasic seeding, with two spinach seeds per planting hole. The filling condition of spinach seeds within the seed-metering hole is illustrated on Fig. 14. To meet agronomic requirements for biphasic spinach seeding, the seed-metering hole dimensions were engineered with three geometric constraints(Fig. 14): depth (*H*_*h*_) satisfies *T* ≤ *H*_*h*_ ≤ *L* for single-seed containment; length (*L*_*h*_) maintains *L* ≤ *L*_*h*_≤ *L* + *T* ensuring monoseed longitudinal alignment; width (*D*_*h*_) adheres to 2*T* ≤ *D*_*h*_ ≤ 2*L* permitting dual-seed lateral accommodation – with the complete parameter space formalized in formula (15) using spinach seed dimensions.

15 $$\left\{ \begin{gathered} L \leqslant {L_h} \leqslant L+T \hfill \\ T \leqslant {H_h} \leqslant L \hfill \\ 2T \leqslant {D_h} \leqslant 2L \hfill \\ \end{gathered} \right.$$

where *L*_*h*_ is the length of seed-metering hole (mm); *H*_*h*_ is the depth of seed-metering hole (mm); *D*_*h*_ is the width of seed-metering hole (mm).

**Fig. 15:**
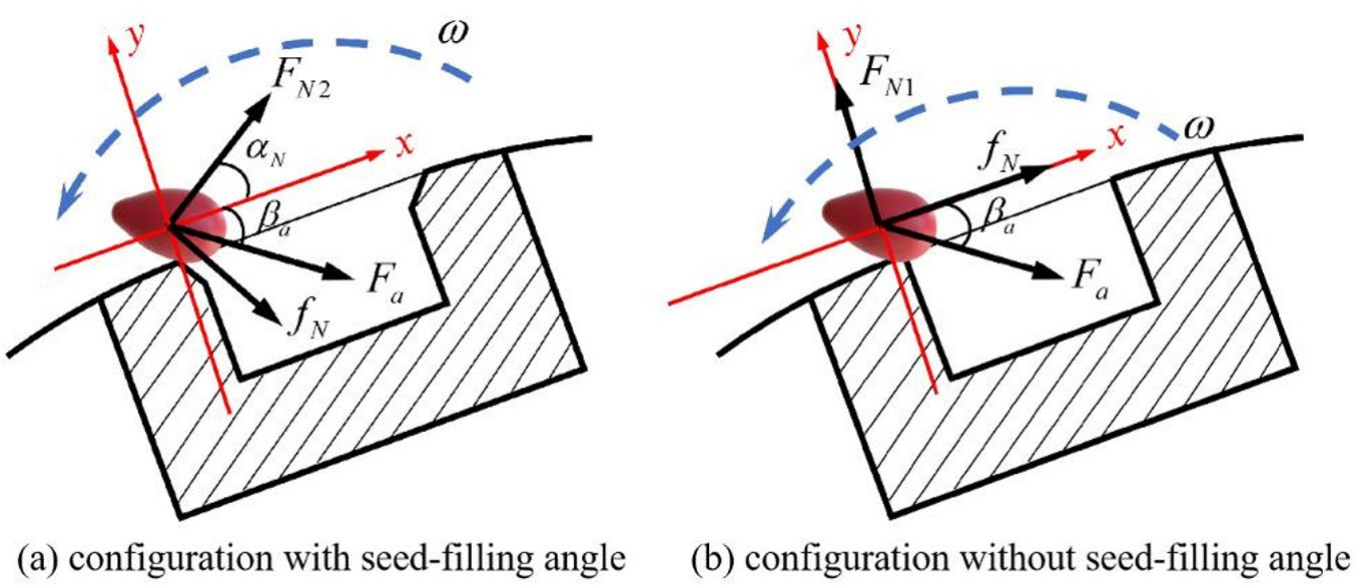
Force analysis during seed filling in the seed-metering wheel.

To investigate the effect of the seed-filling angle on seed-metering performance, a mechanical analysis was conducted on seeds positioned at the edge of the seed-metering hole. As shown in Fig. 15a, a local coordinate system was proved at the seed’s center of mass: the x-axis aligned with the tangential rotation direction of the seed-metering wheel, and the y-axis oriented along the normal direction of the wheel surface. This framework enables the quantitative analysis of seed dynamics during filling.

Comparing scenarios with and without a seed-filling angle reveals a substantial difference in the normal and frictional forces exerted by the seed-metering hole edge on the seeds. Therefore, only these two forces are explicitly labeled in Fig. 15b, while the resultant of other forces is represented as *F*_*a*_. The force analysis for both cases is mathematically expressed in formula (16):

16 $$\left\{ \begin{gathered} {F_y}={F_a}\cos {\beta _a} - {F_{N1}} \hfill \\ {F_{y\theta }}={F_a}\cos {\beta _a} - {F_{N2}}\sin \theta +{f_N}\cos \theta \hfill \\ {F_{y\theta }} - {F_y}={F_{N1}}+{f_N}\cos \theta - {F_{N2}}\sin \theta \hfill \\ \end{gathered} \right.$$

where *F*_*y*_ is the force component along the -y axis on the seed at *θ* = 0° of the seed-metering wheel (N); *F*_*a*_ is the other resultant force on the seed (N); $\:{\beta\:}_{a}$ is the angle between the resultant force Fa and the x-axis in the *xy*-plane (°); F_*N*1_ is the normal contact force exerted by the seed-metering hole edge on the seed at *θ* = 0° (N); *F*_*yθ*_ is the force component along the negative y-axis acting on the seed under seed-filling angle (*θ*) of the seed-metering wheel (N); *F*_*N*2_ is the normal contact force from the seed-metering hole edge on the seed under seed-filling angle(*θ*) (N); *θ* is the seed-filling angle of the seed-metering device (°); *f*_*N*_ is the tangential frictional force exerted by the seed-metering hole edge on the seed(N).

The force component in the negative y-direction facilitates the entry of seeds into the seed-metering hole. Since the difference *F*_*yθ*_-*F*_*y*_ yields consistently positive values, the presence of the seed-filling angle significantly facilitates seed entry into the seed-metering hole. Consequently, seed-metering holes with optimized filling angles exhibit enhanced filling performance. Empirical studies have established that the optimal filling angle ranges from 20° to 60°.

This study appointed the seed-metering hole’s depth (*H*_*h*_), length (*L*_*h*_), width (*D*_*h*_), and seed-filling angle (*θ*) as experimental factors. Design ranges and test levels were established according to Table 5, with the simulated qualified seeding rate (calculated via formula (14)) serving as the response variable. A Box-Behnken design was adopted to conduct simulated seeding tests, aiming to further analyze the influence of numerous factors and complete the optimization of dimensional parameters.

**Table 5:** Experimental parameters and levels of Box-Behnken design for simulated seeding tests.

Experimental Parameter	level		
	Low (− 1)	Middle (0)	High (+ 1)
Seed-metering hole depth *X*_7_/mm	2.14	2.765	3.39
Seed-metering hole length *X*_8_/mm	3.39	4.46	5.53
Seed-metering hole width *X*_9_/mm	4.28	5.53	6.78
Seed-filling angle *X*_10_/ (°)	20	40	60

### Seed-metering device verification bench test

**Fig. 16:**
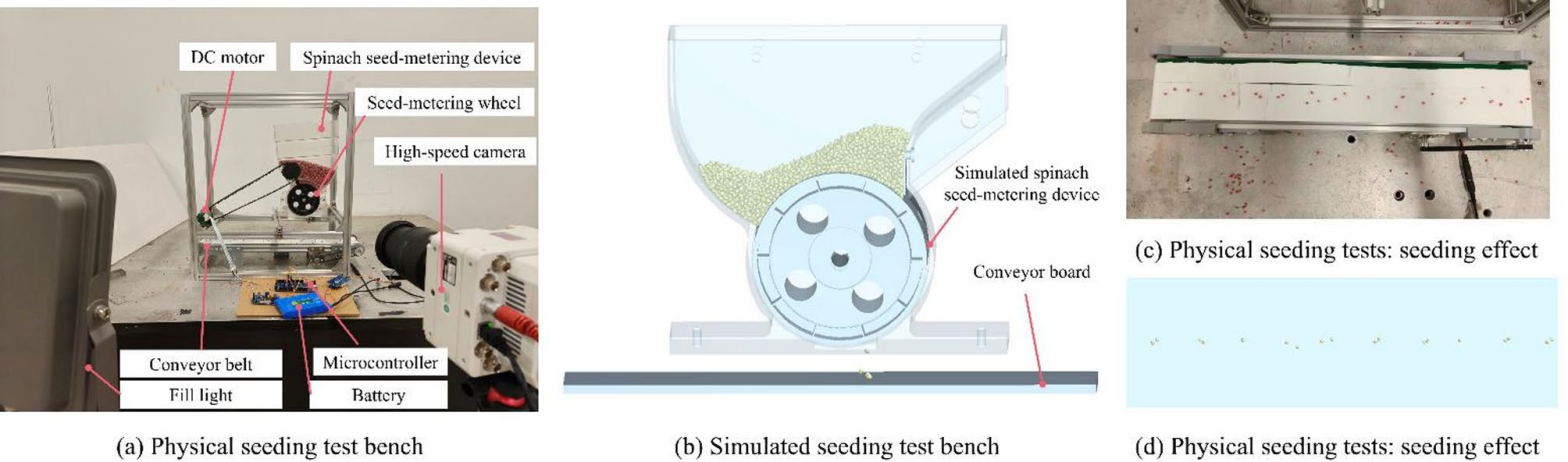
Physical and simulated seeding test bench.

Based on optimized parameters, this study constructed physical and simulated seeding test benches to further verify the rationality and practicability of the spinach seed-metering device design. The physical seeding test bench was composed of a physical spinach seed-metering device, a high-speed camera, a conveyor belt, a fill light, and a storage battery. The simulated seeding test bench was composed of a simulated spinach seed-metering device model and a conveyor board. The physical and simulated seeding test bench is shown on Fig. 16.

Simulated and physical seeding experiments were conducted at three seeding speeds (5r/min, 10r/min, and 15r/min). Performance metrics were analyzed, including seed flow rate, seed damage percentage, single-seed deficit rate, empty hole rate, multi-seed excess rate, missed-seeding rate, qualified seeding rate, seed spacing, intra-hole seed spacing, and seed-metering deviation.

#### Physical seeding test

The physical seeding test of spinach seeds was conducted on the physical seeding test bench, following this procedure: A high-speed camera was used to capture images of the seed-metering device during normal operation, with data acquisition repeated 10 times. From video analysis, the following parameters were obtained: the total normal seeding time (*t*_*p*_), the number of discharged seeds (*n*_*p*_), the number of discharged broken seeds (*n*_*d*_), and the total number of seed-metering holes (*n*_0_)—which was further categorized into holes containing 1 seed (*n*_1_), 0 seeds (*n*_2_), 3 or more seeds (*n*_3_) and 2 seeds (*n*_4_).

**Fig. 17:**
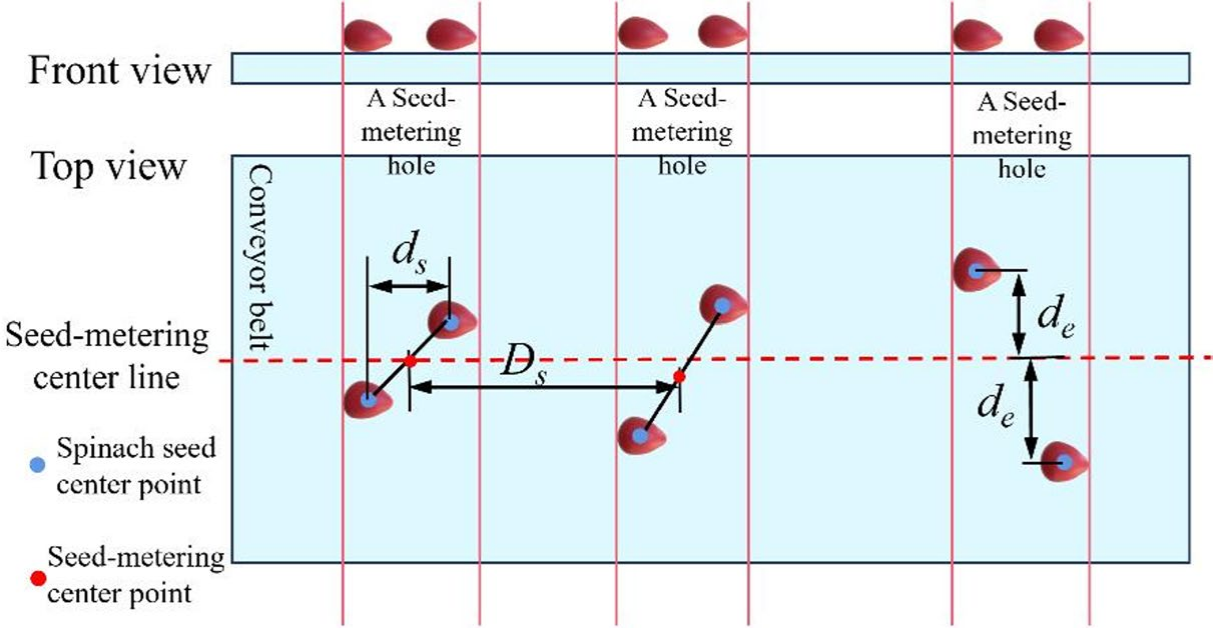
Determination process of seed spacing, intra-hole seed spacing, and seed-metering deviation.

Additionally, a conveyor belt was positioned beneath the seed-metering device; its surface was covered with foam tape, and it operated at a speed of 0.1 m/s. Spinach seeds discharged by the metering-device fell smoothly onto the foam tape, preventing collisions between seeds. A vernier caliper was then used to measure two types of spacing: the horizontal spacing between two seeds in the same hole (defined as physical intra-hole seed spacing) and the horizontal distance between the centers of seeds in adjacent holes (defined as physical seed spacing). Furthermore, the distance between each spinach seed and the central line of the seed-metering device was recorded as physical seed-metering deviation. This entire measurement process—including spacing measurement and seed-metering deviation recording—was repeated 10 times, and the determination process is shown in Fig. 17.

Based on the measured parameters and determined values, the following physical seeding indicators were calculated: physical seed flow rate (*S*_*p*_), physical seed damage percentage (*Q*_*d*_), physical single-seed deficit rate (*Q*_*p*_), physical empty hole rate (*Q*_*e*_), physical multi-seed excess rate (*Q*_*m*_), physical qualified seeding rate (*Q*), physical seed spacing (*D*_*s*_), physical intra-hole seed spacing (*d*_*s*_), and physical seed-metering deviation (*d*_*e*_). The physical missed-seeding rate equals the physical single-seed deficit rate plus the physical single-seed deficit rate.

17 $${S_p}=\frac{{{n_p}}}{{{t_p}}}$$

where *S*_*p*_ is the physical seed flow rate (seeds/s); *n*_*p*_ is the number of seeds discharged (seed); *t*_*p*_ is the total time of normal seeding (s).

18 $${Q_d}=\frac{{{n_d}}}{{{t_p}}} \times 100\%$$

where *Q*_*d*_ is the physical seed damage percentage (%); *n*_*p*_ is the number of seeds discharged (seed); *t*_*p*_ is the total time of normal seeding (s).

19 $${Q_p}=\frac{{{n_1}}}{{{n_0}}} \times 100\%$$

where *Q*_*p*_ is the physical single-seed deficit rate (%); *n*_1_ is the number of seed-metering holes with one seed, *n*_0_ is the total number of seed-metering holes.

20 $${Q_e}=\frac{{{n_2}}}{{{n_0}}} \times 100\%$$

where *Qe* is the physical single-seed deficit rate (%); *n*_2_ is the number of seed-metering holes with 0 seeds.

21 $${Q_m}=\frac{{{n_3}}}{{{n_0}}} \times 100\%$$

where *Q*_*m*_ is the physical multi-seed excess rate (%); *n*_3_ is the number of seed-metering holes with 3 or more seeds.

22 $$Q=\frac{{{n_4}}}{{{n_0}}} \times 100\%$$

where *Q* is the physical qualified seeding rate; *n*_4_ is the number of seed-metering holes with 2 seeds.

#### Simulated seeding test

A simulated seeding test bench was set up in EDEM 2022. After conducting the simulated seeding test, the post-processing interface of EDEM 2022 was used to measure parameters consistent with those in the physical seeding test. These parameters included simulated seed flow rate (*S*_*ps*_), simulated l single-seed deficit rate (*Q*_*ps*_), simulated empty hole rate (*Q*_*es*_), simulated multi-seed excess rate (*Q*_*ms*_), simulated qualified seeding rate (*Q*_*s*_), simulated seed spacing (*D*_*ss*_), simulated intra-hole seed spacing (*d*_*ss*_), and simulated seed-metering deviation (*d*_*es*_).

The process for measuring simulated seed spacing, simulated inter-hole seed spacing, and simulated seed-metering deviation was as follows: In the simulated seeding test bench, a simulated conveyor belt was created beneath the seed-metering device model. The conveyor belt was set to move horizontally at 0.1 m/s. To prevent bouncing or sliding between spinach seed DEM models and the simulated conveyor belt, contact parameters between the two were defined: coefficient of restitution: 0.0001, coefficient of static friction: 1, and coefficient of rolling friction: 1. After completing the simulated seeding process, the “Ruler” function in EDEM 2022’s Tools menu was used to measure simulated seed spacing, simulated intra-hole seed spacing, and simulated seed-metering deviation. This simulated test was repeated 10 times.

## Results and discussion

### Results of physical properties measurement of spinach seeds

Based on prior measurements of the physical properties of spinach seeds, the results are summarized in Table 6.

**Table 6:** Results of physical parameters of spinach seeds.

Physical parameter	Unit	Value
Length × Width × Thickness	mm	3.68 ± 0.40 × 3.24 ± 0.30 × 2.24 ± 0.30
Equivalent particle size	mm	2.99 ± 0.16
Sphericity	%	81.20 ± 4.14
Density	kg m^-3^	1013.89 ± 10.78
Poisson’s ratio		0.32 ± 0.09
Elastic modulus	MPa	19.44 ± 1.46
Shear modulus	MPa	7.36 ± 0.82

Note: Data in the table are mean ± SD.

The ranges of contact parameters for spinach seeds, obtained from previous experimental determinations, are presented in Table 1.

### Results of calibration of contact parameters between spinach seeds

#### Analysis of Plackett-Burman test results

The Plackett-Burman test was designed using Design-Expert 10 software, establishing 12 experimental groups. Subsequently, the simulated angle of repose for each model was measured. The test design and results are listed in Table 7.

**Table 7:** Plackett-Burman test scheme and results.

NO.	X_1_	X_2_	X_3_	X_4_	X_5_	X_6_	Simulated angle of repose(*R*) / (°)
1	0.20	0.30	0.01	0.65	0.30	0.45	31.74
2	0.20	0.60	0.09	0.30	0.62	0.45	42.10
3	0.20	0.30	0.09	0.30	0.62	0.45	40.31
4	0.20	0.30	0.01	0.30	0.30	0.15	35.50
5	0.20	0.60	0.09	0.65	0.30	0.15	44.57
6	0.20	0.60	0.01	0.65	0.62	0.15	33.98
7	0.50	0.60	0.01	0.65	0.62	0.45	33.36
8	0.50	0.30	0.09	0.65	0.62	0.15	38.80
9	0.50	0.30	0.09	0.65	0.30	0.45	36.36
10	0.50	0.60	0.09	0.30	0.30	0.15	41.07
11	0.50	0.60	0.01	0.30	0.30	0.45	33.94
12	0.50	0.30	0.01	0.30	0.62	0.15	28.25

The experiment was executed, and the results were analyzed through variance analysis, with the findings shown in Table 8. The p-value for the model, which includes each factor and the simulated angle of repose, is below 0.01, indicating that the model effectively captures the relationship between each factor and the simulated angle of repose. The p-values for *X*_1_, *X*_2_, and *X*_3_ are less than 0.05, suggesting that these factors significantly influence the response variable. In contrast, the p-values for *X*_4_, *X*_5_, and *X*_6_ are greater than 0.05, indicating that these factors have no significant effect on the response. Consequently, middle-level values for *X*_4_, *X*_5_, and *X*_6_ can be used in subsequent analyses. To obtain precise values for factors *X*_1_, *X*_2_, and *X*_3_, further testing using the BBD is required to complete the determination of the corresponding parameters.

**Table 8:** Variance analysis for Plackett-Burman test results.

Source	Sum of squares	Degree of freedom	Mean Square	F-value	*p*-value
Model	234.81	6	39.14	11.83	0.0079**
*X*_1_	22.47	1	22.47	6.79	0.0479*
*X*_2_	27.18	1	27.18	8.22	0.0351*
*X*_3_	179.72	1	179.72	54.33	0.0007**
*X*_4_	0.4641	1	0.4641	0.1403	0.7233
*X*_5_	3.39	1	3.39	1.03	0.3577
*X*_6_	1.58	1	1.58	0.4788	0.5198
Residual	16.54	5	3.31		
Cor total	251.35	11			

Note: * denotes that the effect of this item is significant (p-value < 0.05); ** denotes that the impact of this item is extremely significant (p-value < 0.01).

#### Analysis of Box-Behnken design test results

The Box-Behnken Design test was developed using Design-Expert 10 software, and the simulated angles of repose were obtained as shown in Table 9.

**Table 9:** Box-Behnken design test scheme and results.

No.	X_1_	X_2_	X_3_	Simulated angle of repose (*R*_S_) / (°)
1	0.35	0.45	0.05	38.64
2	0.35	0.3	0.01	31.78
3	0.2	0.6	0.05	37.54
4	0.35	0.3	0.09	38.25
5	0.35	0.6	0.09	40.29
6	0.35	0.45	0.05	38.64
7	0.2	0.45	0.01	35.64
8	0.5	0.45	0.09	44.77
9	0.5	0.6	0.05	40.38
10	0.35	0.45	0.05	38.93
11	0.35	0.45	0.05	37.00
12	0.5	0.3	0.05	36.82
13	0.35	0.6	0.01	35.39
14	0.2	0.45	0.09	41.39
15	0.5	0.45	0.01	35.44
16	0.35	0.45	0.05	39.13
17	0.2	0.3	0.05	36.39

Variance analysis test results were conducted using Design-Expert 10 software, shown in Table 10. The analysis reveals that the p-value for the model, which includes test variables *X*_1_, *X*_2_, *X*_3_, and the simulated angle of repose, is below 0.01, indicating that the model effectively captures the correlation between the factors and the response variable. The p-value for the lack of fitness is greater than 0.05, which is not significant, suggesting that the model fits well. The p-values for *X*_1_, *X*_2_, and *X*_3_ are all < 0.05, indicating they significantly influence the simulated angle of repose. These results are consistent with the previous Plackett-Burman test. Based on the F-values of *X*_1_, *X*_2_, *X*_3_, the order of significance for each factor on the response variable is as follows: *X*_3_ > *X*_2_ > *X*_1_. The quadratic Eq. (23) relating to experimental factors *X*_1_, *X*_2_, *X*_3_, the simulated angle of repose (*R*_*S*_) was obtained:

23 $$\begin{gathered} {R_S}=38.47+0.8063{X_1}+1.30{X_2}+3.31{X_3}+0.6025{X_1}{X_2}+0.8950{X_1}{X_3} - \hfill \\ {\text{ }}0.3925{X_2}{X_3}+1.10{X_1}^{2} - 1.78{X_2}^{2} - 0.2565{X_3}^{2} \hfill \\ \end{gathered}$$

**Table 10:** Analysis of variance of Box-Behnken design test results.

Source	Sum of squares	Degree of freedom	Mean square	F-value	*p*-value
Model	129.37	9	14.37	21.42	0.0003**
*X*_1_	5.20	1	5.20	7.75	0.0272*
*X*_2_	13.42	1	13.42	19.99	0.0029**
*X*_3_	87.45	1	87.45	130.3	< 0.0001
*X*_1_ *X*_2_	1.45	1	1.45	2.16	0.1848
*X*_1_ *X*_3_	3.20	1	3.20	4.77	0.0652
*X*_2_ *X*_3_	0.6162	1	0.6162	0.9182	0.3699
*X*_1_²	5.08	1	5.08	7.57	0.0284*
*X*_2_²	13.4	1	13.40	19.97	0.0029**
*X*_3_²	0.277	1	0.277	0.4128	0.541
Residual	4.70	7	0.6711		
Lack of Fit	1.83	3	0.6107	0.8523	0.5336
Pure Error	2.87	4	0.7165		
Cor Total	134.07	16			

Note: * denotes that the effect of this item is significant (p-value < 0.05); ** denotes that the impact of this item is extremely significant (p-value < 0.01).

where *R*_*S*_ is the simulated angle of repose.

#### Determination of contact parameters

The average physical angle of repose (*R* = 38.27°) and its standard deviation (0.79°) were determined. The objective function is that the value of the quadratic equation for the simulated repose angle *R*_*S*_ (with factors *X*_1_, *X*_2_, and *X*_3_ as variables) approaches the mean physical repose angle *R*, with parameter constraints and other details shown in the formula (24). Accordingly, the average physical angle of repose (*R* = 38.27°) was selected as the target value for this optimization. Subsequently, optimization was performed via Design-Expert 10 software using the quadratic model, through which the coefficients for restitution (*X*_1_), static friction (*X*_2_), and rolling friction (*X*_3_) between spinach seeds were determined to be 0.385, 0.481, and 0.042, respectively.

24 $$\left\{ \begin{gathered} obj:{R_S} \to R=38.27^\circ \hfill \\ s.t\left\{ \begin{gathered} 0.2 \leqslant {X_1} \leqslant 0.5 \hfill \\ 0.3 \leqslant {X_2} \leqslant 0.6 \hfill \\ 0.01 \leqslant {X_3} \leqslant 0.09 \hfill \\ \end{gathered} \right. \hfill \\ \end{gathered} \right.$$

Based on ten simulated repose angle verification tests, the mean simulated repose angle was 37.95° with a standard deviation of 0.71°, exhibiting a maximum deviation of only 0.8% from the mean value of the physical repose angle test. The root means square error (RMSE) between the physical mean repose angle (*R* = 38.27°) and the ten simulated results was 0.75°, indicating a low overall deviation between the simulated and the physical experiment. From the perspective of error quantification, these results demonstrate that the simulated reliably reproduces the average characteristics of the physical repose angle.

A statistical analysis was conducted on the physical angle of repose test and the simulated verification test. The K–S test (p-value > 0.05) indicated that both datasets approximately followed a normal distribution, while the Welch’s t-test (p-value > 0.05) showed no significant difference in the mean repose angles between the two groups. These results confirm that the calibrated contact parameters of the spinach seed DEM model are in good agreement with the physical experiment, demonstrating the reliability and validity of the model at the statistical distribution level.

### Results of calibration of spinach seed-contact material contact parameters

According to the test factors and levels in Table 4, the BBD was adopted to conduct the simulated fluidity test. The test results correspond to different combinations of test factors and the simulated mass flow rate were obtained, as shown in Table 11.

**Table 11:** Results of the simulated fluidity test.

No.	X_4_	X_5_	X_6_	Simulated mass flow rate (V_fs_)/(g/s)
1	0.3	0.3	0.3	23.21
2	0.65	0.3	0.3	22.64
3	0.3	0.62	0.3	18.77
4	0.65	0.62	0.3	19.05
5	0.3	0.46	0.15	21.03
6	0.65	0.46	0.15	22.19
7	0.3	0.46	0.45	20.96
8	0.65	0.46	0.45	20.14
9	0.475	0.3	0.15	22.75
10	0.475	0.62	0.15	17.98
11	0.475	0.3	0.45	21.51
12	0.475	0.62	0.45	17.29
13	0.475	0.46	0.3	20.96
14	0.475	0.46	0.3	21.33
15	0.475	0.46	0.3	21.89
16	0.475	0.46	0.3	21.39
17	0.475	0.46	0.3	21.65

The Design-Expert 10 software was used to perform variance analysis on the experimental results, and the variance analysis table (Table 12) was obtained. The results of the variance analysis are as follows: The p-value for the correlation model between the experimental variables *X*_4_, *X*_5_, *X*_6_ and the simulated mass flow rate is less than 0.001, indicating that the model effectively reflects the relationship between the factors and the response variable (simulated mass flow rate). The p-value for the “lack of fit” is greater than 0.05, indicating no significant difference, and thus the model fits well. Among the experimental variables, the p-values for *X*_5_ and *X*_6_ are both less than 0.05, suggesting that these two variables have a significant impact on the simulated mass flow rate, while *X*_4_ does not significantly affect the simulated mass flow rate. Based on the F-values of the factors, the order of significance for the response variable (simulated mass flow rate) is as follows: *X*_5_ > *X*_6_ > *X*_4_. Finally, the quadratic equation for the relationship between the experimental variables *X*_4_, *X*_5_, *X*_6_ and the simulated mass flow rate is given in formula (25).

25 $$\begin{gathered} {V_f}_{s}=20.87 - 8.21{X_4}+12.36{X_5}+21.60{X_6}+7.59{X_4}{X_5} - 18.86{X_4}{X_6} \hfill \\ {\text{ }}+5.73{X_5}{X_6}+10.96{X_4}^{2} - 33.67{X_5}^{2} - 31.09{X_6}^{2} \hfill \\ \end{gathered}$$

**Table 12:** Analysis of variance of Box-Behnken design test results.

Source	Sum of squares	Degree of freedom	Mean square	F-value	*p*-value
Model	45.23	9	5.03	53.23	< 0.0001**
*X*_4_	0.0003	1	0.0003	0.0033	0.9557
*X*_5_	36.21	1	36.21	383.55	< 0.0001**
*X*_6_	2.05	1	2.05	21.72	0.0023**
*X*_4_ *X*_5_	0.1806	1	0.1806	1.91	0.2091
*X*_4_ *X*_6_	0.9801	1	0.9801	10.38	0.0146*
*X*_5_ *X*_6_	0.0756	1	0.0756	0.8011	0.4005
*X*_4_²	0.4739	1	0.4739	5.02	0.0600*
*X*_5_²	3.13	1	3.13	33.14	0.0007**
*X*_6_²	2.06	1	2.06	21.82	0.0023**
Residual	0.6608	7	0.0944		
Lack of Fit	0.1693	3	0.0564	0.4593	0.7256
Pure Error	0.4915	4	0.1229		
Cor Total	45.89	16			

Note: * denotes that the effect of this item is significant (p-value < 0.05); ** denotes that the impact of this item is extremely significant (p-value < 0.01).

The mean physical mass flow rate obtained from the physical fluidity test was 18.60 g/s, with a standard deviation of 0.70 g/s. The objective function was formulated to minimize the discrepancy between the quadratic equation (formula (25)) for the model parameters *X*_4_, *X*_5_, and *X*_6_, and the simulated mass flow rate, aiming for the value to approach the mean physical mass flow rate. The constraints applied were as follows: *X*_4_ was within the range of [0.3,0.6], *X*_5_ ranged from [0.3, 0.62], and *X*_6_ varied between [0.15,0.45], as specified in formula (26). Using the “Numerical” function in the “Optimization” module of Design-Expert software, the optimized values for the coefficients of restitution (*X*_4_), static friction (*X*_5_), and rolling friction (*X*_6_) between spinach seeds and PVC plate were determined to be 0.339, 0.600, and 0.408, respectively.

26 $$\left\{ \begin{gathered} obj:{V_{fs}} \to {V_f}=18.60{\text{ }}g/s \hfill \\ s.t\left\{ \begin{gathered} 0.3 \leqslant {X_3} \leqslant 0.6 \hfill \\ 0.3 \leqslant {X_4} \leqslant 0.62 \hfill \\ 0.15 \leqslant {X_6} \leqslant 0.45 \hfill \\ \end{gathered} \right. \hfill \\ \end{gathered} \right.$$

To verify the accuracy of the discrete element method (DEM) model for spinach seeds, the parameters calibrated in the previous section were imported into EDEM 2022 software to conduct simulated fluidity tests. The tests yielded an average simulated mass flow rate of 18.17 g/s with a standard deviation of 0.45 g/s; the relative deviation between this average simulated value and the average physical mass flow rate was 2.31%. Additionally, the RMSE between the average physical mass flow rate (*V*_*f*_ = 18.603 g/s) and the 10 sets of simulated mass flow rate data was 0.431 g/s, which indicates that the overall deviation between the simulated results and physical experimental data is small.

Subsequently, statistical analysis was performed on the data from the physical fluidity tests and simulated verification tests. First, the Kolmogorov-Smirnov (K-S) test (p-value > 0.05) confirmed that both datasets approximately followed a normal distribution—a fundamental prerequisite for conducting the independent samples Welch’s t-test. Further, the independent samples Welch’s t-test (p-value > 0.05) revealed no significant difference in the average mass flow rate between the physical and simulated groups. These results demonstrate that the contact parameters of the calibrated spinach seed DEM model are in good agreement with physical experimental outcomes, thereby verifying the model’s reliability and effectiveness at the statistical distribution level.

### Results of parameter validation of the spinach seed DEM model

To further validate the accuracy of parameters calibrated via the repose angle and fluidity tests, this study compares the results of physical and simulated bulk density tests. The test results are presented in Table 13. Analysis indicates that under different vertical distances between the funnel outlet and the beaker’s rim, the mean values of the physical and simulated bulk density tests are close, with a mean relative error of less than 7% and a coefficient of variation (CV) of bulk density below 3%. These findings demonstrate that the data from both physical and simulated bulk density accumulation tests exhibit high accuracy and stability.

**Table 13:** Result of physical and simulated bulk density tests.

Vertical distance	Group	Mean ± SD	CV (coefficient of variation)	Mean deviation
50 mm	Physical	0.558 ± 0.014	2.57%	3.04%
	Simulated	0.575 ± 0.015	2.67%	
100 mm	Physical	0.580 ± 0.015	2.56%	2.93%
	Simulated	0.597 ± 0.011	1.89%	
150 mm	Physical	0.585 ± 0.015	2.57%	3.66%
	Simulated	0.606 ± 0.015	2.54%	

Note: Data in the table are mean ± SD.

K-S tests and Welch’s t-tests were performed on the data from simulated and physical bulk density tests. The results showed that under all three vertical distance conditions, the p-values of both tests were greater than 0.05: specifically, the K-S test indicated that the probability distributions of the simulated and physical bulk density data were approximately consistent, while the Welch’s t-test revealed no statistically significant difference in the means between the two groups of data. This result confirms that the outcomes of the simulated bulk density tests and physical bulk density tests exhibit a high degree of consistency.

Based on the above analysis, the discrete element model established in this study exhibits high accuracy and can effectively simulate interactions both between spinach seeds and between spinach seeds and contact materials.

Both physical and simulated bulk densities increase gradually with increasing vertical distance. This is because a greater vertical distance enhances the dispersion degree of seeds falling into the beaker, thereby reducing inter-seed gaps and increasing the number of seeds contained in the beaker. At each vertical distance, the physical bulk density is smaller than the simulated bulk density. This discrepancy arises because: (1) the spinach seed DEM model in the simulation does not fully replicate the complex morphology of actual spinach seeds; (2) during physical movement, the spinach seeds undergo plastic deformation, which leads to more regular movement patterns; and (3) spinach seeds may carry like charges during movement. As a result, the spatial gaps between seed models in the simulation are smaller than those between actual spinach seeds in the physical test, leading to a higher simulated bulk density compared to the physical bulk density.

### Results of optimization and design for spinach seed-metering device

#### Results of structural design and performance validation

**Fig. 18:**
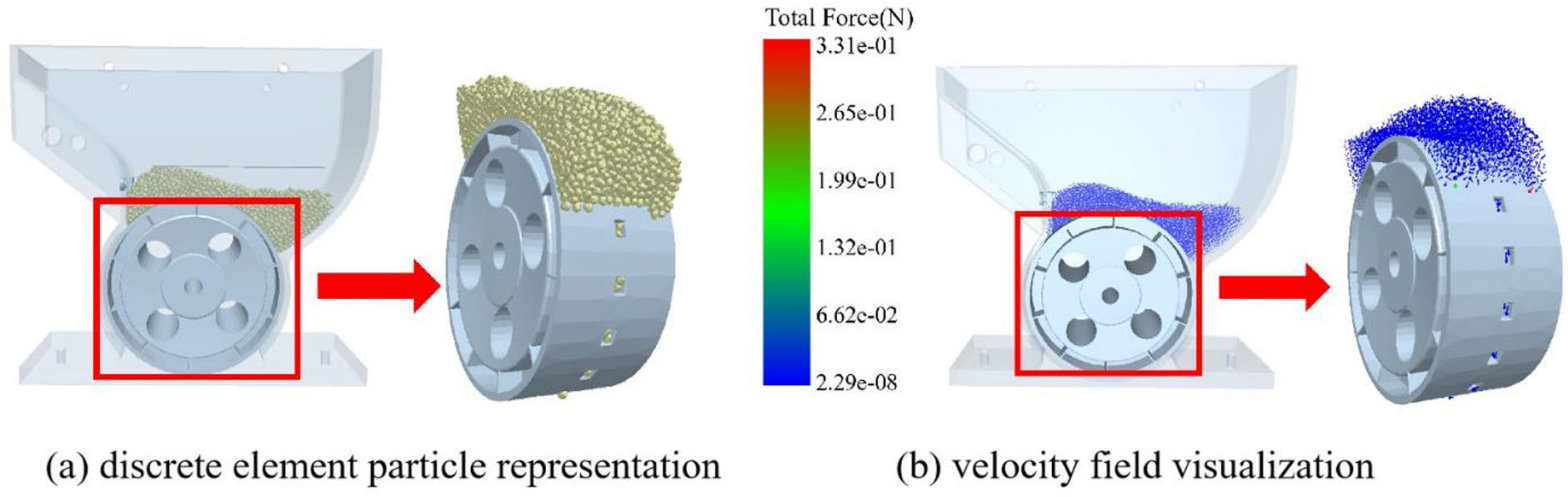
Spinach seed-metering process simulation.

Seeding process simulations were carried out in EDEM 2022. The post-processor extracted particle trajectories of spinach seeds from the DEM models (Fig. 18). Analysis revealed synchronous seed movement with the seed-metering wheel, confirming the effectiveness of the device’s design. The seeding performance of the preliminary design of the seed-metering device is as follows: the mean value of the simulated qualified seeding rate was 83.51%, with a standard deviation of 0.49%. This seed-metering device can initially meet the spinach seeding requirements.

#### Results of seed-metering wheel optimization design

This study conducted discrete element simulation experiments of a spinach seed-metering device based on the Box-Behnken Design experimental scheme. The test result data (Table 14) were processed and analyzed for variance using Design-Expert 10 software, with the analytical results presented in Table 15.

**Table 14:** Schemes and results of BBD test for simulated seeding tests.

No.	X_7_ (mm)	X_8_ (mm)	X_9_ (mm)	X_10_ (°)	Qs (%)
1	2.765	4.46	5.53	40	87.00
2	2.765	5.53	5.53	60	58.00
3	2.765	4.46	5.53	40	96.00
4	3.390	4.46	4.28	40	45.00
5	2.765	5.53	5.53	20	51.00
6	2.140	4.46	6.78	40	1.00
7	2.765	3.39	5.53	60	85.00
8	2.765	4.46	6.78	20	9.00
9	2.765	3.39	4.28	40	73.00
10	3.390	4.46	5.53	60	58.00
11	2.765	4.46	5.53	40	92.00
12	3.390	5.53	5.53	40	28.00
13	2.140	5.53	5.53	40	40.00
14	2.765	4.46	6.78	60	1.00
15	2.765	5.53	4.28	40	52.00
16	3.390	4.46	6.78	40	3.00
17	2.765	4.46	5.53	40	82.00
18	2.140	4.46	5.53	60	69.00
19	3.390	3.39	5.53	40	49.00
20	2.765	5.53	6.78	40	1.00
21	2.765	4.46	4.28	20	69.00
22	2.140	4.46	4.28	40	66.00
23	2.765	3.39	6.78	40	10.00
24	2.140	3.39	5.53	40	81.00
25	2.765	4.46	4.28	60	80.00
26	2.765	4.46	5.53	40	92.00
27	2.765	3.39	5.53	20	81.00
28	2.140	4.46	5.53	20	63.00
29	3.390	4.46	5.53	20	59.00

**Table 15:** Analysis of variance of Box-Behnken design test results.

Source	Sum of squares	Degree of freedom	Mean square	F-value	*p*-value
Model	26067.52	14	1861.97	42.2	< 0.0001**
*X*_7_	507.00	1	507.00	11.49	0.0044**
*X*_8_	1850.08	1	1850.08	41.93	< 0.0001**
*X*_9_	10800.00	1	10800.00	244.77	< 0.0001**
*X*_10_	30.08	1	30.08	0.6818	0.4228
*X*_7_ *X*_8_	100.00	1	100.00	2.27	0.1544
*X*_7_ *X*_9_	132.25	1	132.25	3	0.1054
*X*_7_ *X*_10_	12.25	1	12.25	0.2776	0.6065
*X*_8_ *X*_9_	36.00	1	36.00	0.8159	0.3817
*X*_8_ *X*_10_	2.25	1	2.25	0.051	0.8246
*X*_9_ *X*_10_	90.25	1	90.25	2.05	0.1746
*X*_7_²	3087.35	1	3087.35	69.97	< 0.0001**
*X*_8_²	1648.45	1	1648.45	37.36	< 0.0001**
*X*_9_²	10806.49	1	10806.49	244.92	< 0.0001**
*X*_10_²	290.45	1	290.45	6.58	0.0224*
Residual	617.72	14	44.12		
Lack of Fit	500.92	10	50.09	1.72	0.3178
Pure Error	116.80	4	29.20		
Cor Total	26685.24	28			

Note: * denotes that the effect of this item is significant (p-value < 0.05); ** denotes that the impact of this item is extremely significant (p-value < 0.01).

The simulated qualified seeding rate (*Q*_*s*_) data presented in Table 14 are derived from simulated seeding experiments. The effects of four key design parameters—hole depth (*X*_7_), hole length (*X*_8_), hole width (*X*_9_), and seed-filling angle (*X*_10_)—on the simulated qualified seeding rate of simulated seeding were analyzed using Design-Expert 10. A quadratic regression model (formula (27)) was developed to describe the relationship between each factor and the simulated qualified seeding rate of simulated seeding. The coefficient of determination (*R*^2^) for the model is 0.9769, and the adjusted *R*^2^ is 0.9537, both of which are close to 1, indicating that the model accurately predicts the simulated qualified seeding rate of simulated seeding. The model’s Adequate Precision is greater than 4, which suggests that it can effectively distinguish between signal and noise, making it suitable for optimizing subsequent design parameters.

27 $$\begin{gathered} {Q_s}=89.80 - 6.50{X_7} - 12.42{X_8} - 30.00{X_9}+1.58{X_{10}}+5.00{X_7}{X_8}+ \hfill \\ {\text{ 5}}{\mathrm{.75}}{X_7}{X_9} - 1.75{X_7}{X_{10}}+3.00{X_8}{X_9} - 0.75{X_8}{X_{10}} - \hfill \\ {\text{ }}{4.75_9}{X_{10}} - 21.82{X_7}^{2} - 15.94{X_8}^{2} - 40.82{X_9}^{2} - 6.69{X_{10}}^{2} \hfill \\ \end{gathered}$$

According to the analysis of variance (Table 15), the p-value for the entire model is less than 0.01, while the p-value for the lack of fit is greater than 0.1, indicating that the model fits well. From the p-values of each factor, it is evident that *X*_7_, *X*_8_, *X*_9_, *X*_7_^2^, *X*_8_^2^, and *X*_9_^2^, *X*_10_^2^ have an extremely significant effect on the simulated qualified seeding rate, while the effect of the other factors on the qualification coefficient is not significant.

**Fig. 19:**
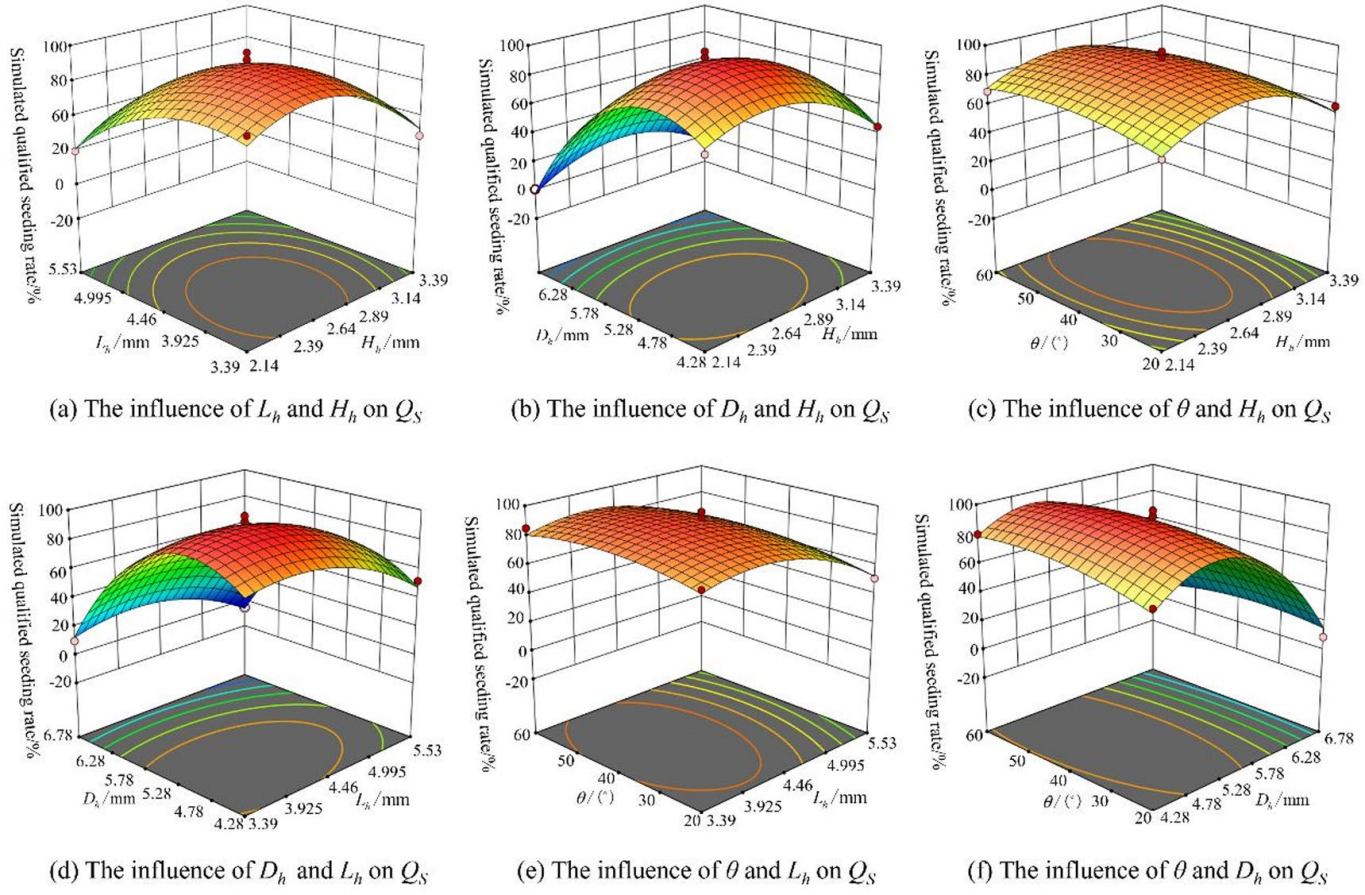
Response surface of the interaction effects of various factors on the simulated qualified seeding rate.

The effects of various factors on the simulated qualified seeding rate (*Q*_*s*_) were further analyzed. As shown in Fig. 19a, when *H*_*h*_ or *L*_*h*_ remained unchanged, *Q*_*s*_ initially increased and then decreased with the increase of *L*_*h*_ and *H*_*h*_. The change in *Q*_*s*_ with *L*_*h*_ was more pronounced, indicating that *L*_*h*_ had a more significant effect on *Q*_*s*_. Figure 19b illustrates that when *D*_*h*_ or *H*_*h*_ remained unchanged, *Q*_*s*_ first increased and then decreased as *H*_*h*_ and *D*_*h*_ increased. The change in *Q*_*s*_ with *D*_*h*_ was more significant, so *D*_*h*_ had a greater impact on *Q*_*s*_. As shown in Fig. 19c, when *θ* or *H*_*h*_ remained unchanged, *Q*_*s*_ increased and then decreased with the increase of *H*_*h*_ and *θ*, with the change in *Q*_*s*_ being more pronounced with *H*_*h*_. Thus, *H*_*h*_ had a more significant influence on *Q*_*s*_. Figure 19d shows that when *D*_*h*_ or *L*_*h*_ remained unchanged, *Q*_*s*_ initially increased and then decreased with the increase of *L*_*h*_ and *D*_*h*_. The change in *Q*_*s*_ with *D*_*h*_ was more significant, indicating that *Q*_*s*_ had a more substantial effect on *Q*_*s*_. Figure 19e demonstrates that when *θ* or *L*_*h*_ remained unchanged, *Q*_*s*_ increased and then decreased with the increase of *L*_*h*_ and *θ*, with *Q*_*s*_ showing more noticeable changes with *L*_*h*_. Therefore, *L*_*h*_ had a more significant effect on *Q*_*s*_. Figure 19f indicates that when *θ* or *D*_*h*_ remained unchanged, *Q*_*s*_ increased and then decreased as *D*_*h*_ and *θ* increased, with *Q*_*s*_ changing more significantly with *D*_*h*_. Thus, *D*_*h*_ had a greater impact on *Q*_*s*_.

Based on the analysis, the sensitivity of the four factors to *Q*_*s*_ is as follows: *D*_*h*_> *L*_*h*_> *H*_*h*_ > *θ*, which is consistent with the F-values of the factors presented in the Table 15.

Based on the quadratic regression model (formula (28)) established using DEM seeding tests., the “Numerical” function in the “Optimization” module of Design-Expert 10 software was used to solve the optimization problem with the objective of maximizing the qualified seeding rate. The constraint conditions were as follows: the value range for *X*_7_ (hole depth) was [2.14 mm, 3.39 mm], for *X*_8_ (hole length) was [3.39 mm, 5.53 mm], for *X*_9_ (hole width) was [4.28 mm, 6.78 mm], and for the seed-filling *X*_10_ was [20°, 60°]. Parameter optimization of the spinach seed-metering device yielded final dimensions *H*_*h*_=2.52 mm, *L*_*h*_=3.67 mm, *D*_*h*_=5.15 mm, and seed-filling angle *θ* = 37.99°, achieving a 96.24% predicted simulated qualified seeding rate at 10 r/min seeding speed.

28 $$\left\{ \begin{gathered} obj:\hbox{max} {Q_s} \hfill \\ s.t\left\{ \begin{gathered} 2.14{\mathrm{mm}} \leqslant {X_7} \leqslant 3.39{\mathrm{mm}} \hfill \\ 3.39{\mathrm{mm}} \leqslant {X_8} \leqslant 5.53{\mathrm{mm}} \hfill \\ 4.28{\mathrm{mm}} \leqslant {X_9} \leqslant 6.78{\mathrm{mm}} \hfill \\ 20^\circ \leqslant {X_{10}} \leqslant 60^\circ \hfill \\ \end{gathered} \right. \hfill \\ \end{gathered} \right.$$

To verify the reliability of the discrete element method (DEM)-based simulated optimization results, the optimized parameters were input into EDEM 2022 to conduct 10 simulated verification tests for seeding. The tests yielded a mean simulated qualified seeding rate of 93.17% with a standard deviation of 1.05%. The relative deviation between this mean index and the predicted seeding qualification index (96.24%) was 3.16%, and the RMSE between the 10 sets of simulated seeding qualified seeding rate and the predicted value was 3.06%. These results indicate that the error between the simulated and predicted simulated qualified seeding rate is small, confirming that the optimization results for the metering device are acceptable.

### Results of seed-metering device verification bench test

This study involved the construction of both a physical and simulated seeding test bench. The physical and simulated seeding tests were conducted to obtain various performance metrics. The mean value, standard deviation, relative deviation (RE) (as shown in Table 16), and coefficient of variation (CV) (as shown in Table 17) for each index were calculated. Subsequently, K-S tests and Welch’s t-tests were performed on the physical and simulated test data.

**Table 16:** The various performance metrics of physical and simulated seeding tests.

Performance metrics	5r/min			10r/min			15r/min		
	Simulated	Physical	RE (%)	Simulated	Physical	RE (%)	Simulated	Physical	RE (%)
Seed flow rate(seed/s)	2.81 ± 0.11	2.69 ± 0.17	4.27	5.87 ± 0.08	5.68 ± 0.12	3.24	8.67 ± 0.34	8.44 ± 0.28	2.65
Single-seed deficit rate (%)	5.49 ± 0.71	5.98 ± 0.91	8.93	3.83 ± 0.78	4.57 ± 0.91	19.32	5.71 ± 0.70	6.65 ± 1.40	16.46
Empty hole rate (%)	1.61 ± 0.33	2.11 ± 0.67	31.06	1.58 ± 0.37	1.76 ± 0.34	11.39	1.67 ± 0.30	2.00 ± 0.42	19.76
Multi-seed excess rate (%)	1.40 ± 0.23	1.56 ± 0.25	11.43	1.42 ± 0.36	1.44 ± 0.31	1.41	1.53 ± 0.26	1.56 ± 0.15	1.96
Qualified seeding rate (%)	91.45 ± 1.02	90.36 ± 1.29	1.19	93.17 ± 1.05	92.23 ± 0.93	1.01	91.09 ± 0.88	89.79 ± 1.53	1.43
Seed spacing (mm)	67.41 ± 2.37	70.60 ± 5.70	4.73	32.26 ± 1.53	33.56 ± 1.31	4.03	23.29 ± 2.11	24.33 ± 1.24	4.47
Intra-hole seed spacing(mm)	2.25 ± 1.36	2.49 ± 0.78	10.67	2.32 ± 0.61	2.65 ± 0.64	14.22	2.17 ± 1.16	2.53 ± 0.72	16.59
Seed-metering deviation (mm)	1.35 ± 0.89	1.50 ± 0.43	11.11	1.41 ± 0.81	1.62 ± 0.48	14.89	1.66 ± 0.50	1.95 ± 0.53	17.47
Seed damage percentage. (%)		3.16 ± 0.20			1.95 ± 0.28			2.60 ± 0.17	

Note: Data in the table are mean ± SD.

**Table 17:** The coefficient of variation (CV) of various evaluation indices for physical and simulated seeding tests.

Performance metrics	5r/min		10r/min		15r/min	
	S.CV	*P*.CV	S.CV	*P*.CV	S.CV	*P*.CV
Seed flow rate(seed/s)	3.86%	6.46%	1.29%	2.09%	3.94%	3.33%
Single-seed deficit rate (%)	12.85%	15.25%	20.44%	19.88%	12.20%	21.09%
Empty hole rate (%)	19.58%	31.78%	23.57%	19.45%	17.97%	21.13%
Multi-seed excess rate (%)	16.20%	16.10%	25.62%	21.60%	16.80%	9.88%
Qualified seeding rate (%)	1.12%	1.42%	1.13%	1.00%	0.96%	1.70%
Seed spacing (mm)	3.52%	8.07%	4.71%	3.91%	9.04%	5.09%
Intra-hole seed spacing(mm)	60.37%	31.21%	26.48%	24.19%	53.52%	28.65%
Seed-metering deviation (mm)	68.12%	28.68%	57.14%	29.62%	29.90%	26.91%
Seed damage percentage. (%)		6.39%		14.35		6.62%

Note: S.CV means the CV of simulated performance metrics, P.CV means the CV of physical performance metrics.

As shown in Table 17, during the initial design of this seed-metering device, we preliminarily determined its parameters: the seed-metering hole depth is 3 mm, with a length of 4.5 mm and a width of 5.5 mm. This serves as the preliminary design of the seed-metering device. Simulated seeding tests were conducted to obtain the simulated qualified seeding rate. Through a series of optimizations, the final optimized parameters of the seed-metering device were obtained. Based on these parameters, the spinach seed-metering device was constructed, and simulated seeding tests were carried out to obtain the optimal simulated qualified seeding rate. In terms of the simulated qualified seeding rate, the finally optimized seed-metering device showed an improvement of 10.02% (increasing from 83.15% to 93.17%).

The relative deviation error between the seed flow rates of the physical and simulated seeding tests is smallest at a speed of 15 r/min. This is because, at higher seeding speeds, the seed flow rate in the physical test closely matches that of the simulated test. Table 16 also reveals that seeding uniformity is low at both 5 r/min and 15 r/min. At the lower speed, the seed-metering device experiences jamming, as indicated by the maximum coefficient of variation for seed flow rate in the physical test at 5 r/min. While higher speeds introduce more disturbances, the coefficient of variation remains below 7% at all speeds, demonstrating that the seed flow rate satisfies agricultural production requirements. Statistical analysis shows that at both 10 r/min and 15 r/min, the K-S test (p-value > 0.05) and Welch’s t-test (p-value > 0.05) reveal no significant difference between the physical and simulated test results. At 5 r/min, although there is a slight difference in data distribution (K-S test p-value = 0.012), the mean difference is not statistically significant (Welch’s t-test p-value = 0.056), confirming that the simulated seeding test accurately replicates the physical seeding process.

As the seeding speed increased, the physical seed damage percentage first rose, then fell, and then rose again; at 10 r/min, the physical seed damage percentage was the lowest. The reasons are: at lower rotational speeds, the physical seed-metering device is prone to jamming, which damages spinach seeds; at higher rotational speeds, the device tends to carry seeds that should not enter the metering holes into the metering process and damage them due to impacts with the device’s inner wall.

The empty hole rate deviation and the coefficient of variation of each test group in the physical and simulated tests were relatively high. This is because the empty hole rate data were small, and small fluctuations would increase the deviation and coefficient of variation. Since the empty hole rates in both physical and simulated seeding tests were small (below 2%), the deviation and coefficient of variation were acceptable. Results of statistical tests showed that: under all seeding speed conditions, the results of the K-S test (p-value > 0.05) and Welch’s t-test (p-value > 0.05) indicated that the data distributions of the physical and simulated tests were similar, and there was no systematic deviation in the mean values of the two datasets.

The deviation in the single-seed deficit rate between the physical and simulated seeding tests increases with the seeding speed of the seed-metering device. This is because, at higher rotation speeds, external disturbances in the physical seeding test become more pronounced. At 10 r/min, the single-seed deficit rate is the lowest in both the physical and simulated tests. Statistical analysis indicates that at all speeds, both the K-S test (p-value > 0.05) and Welch’s t-test (p-value > 0.05) show no significant differences between the data distributions of the physical and simulated tests, confirming that there is no systematic deviation in the mean values.

The multi-seed excess rate deviation between the physical and simulated seeding tests decreases as the seeding speed of the metering device increases. With higher speeds in the physical seeding test, fewer seeds enter the seeding holes, thereby reducing the error between the physical and simulated tests. The multi-seed excess rate is minimal at 10 r/min for both physical and simulated tests. Extreme rotational speeds, either too low or too high, result in seed blockages within the seed-metering holes, leading to an increased multi-seed excess rate. Statistical analysis reveals no significant differences in data distribution between the two test groups (K-S test, p-value > 0.05), and no systematic deviation in the mean values (Welch’s t-test, p-value > 0.05).

The qualified seeding rate deviation for both physical and simulated seeding tests remain small (< 3%) across all rotational speeds (5 r/min, 10 r/min, and 15 r/min), with coefficients of variation below 2%. These results indicate that the performance of both the physical and simulated tests is consistent and stable under the specified conditions. Notably, both tests achieved the highest qualified seeding rate at 10 r/min (physical 92.26%, simulated 93.17%), with exceptionally high data stability. Increasing the seed-metering wheel’s rotational speed raises seed velocity within the filling zone, improving the chance of seeds entering the holes. However, at excessively high speeds, the time for the holes to pass through the filling zone decreases significantly, reducing seed entry and lowering the qualified seeding rate. Therefore, the operational speed of the designed spinach seed-metering device should be maintained below a critical threshold to ensure optimal performance. This highlights that the seed-metering device operates optimally at this speed. Statistical tests show no significant differences in data distribution across the different speeds (K-S test, p-value > 0.05) and no systematic mean deviation (Welch’s t-test, p-value > 0.05), further confirming the high reliability of the discrete element simulation model in predicting the qualified seeding rate.

Regarding seed spacing deviation, no significant difference was observed between the physical and simulated seeding tests at each rotational speed. The deviations remained within a reasonable range, and the coefficient of variation for all test groups was consistently below 8%, reflecting high stability. Notably, the physical test demonstrated the highest stability at 10 r/min (CV = 3.9%). Statistical tests revealed no significant differences in the distribution pattern (K-S test, p-value > 0.05) or mean deviation (Welch’s t-test, p-value > 0.05) between the two groups.

The deviation in intra-hole seed spacing was relatively large in both the physical and simulated seeding tests, with the deviation increasing as the rotational speed increased. This can be attributed to the greater influence of external factors (e.g., vibration, air resistance, and seed discharge collisions) in the physical test, which led to larger particle spacing in the physical holes compared to the simulation. Consequently, the physical test exhibited a higher coefficient of variation for intra-hole spacing, resulting in poorer data stability. Statistical tests showed no significant differences in data distribution (K-S test, p-value > 0.05) or mean deviation (Welch’s t-test, p-value > 0.05) between the two groups.

The seed-metering deviation between the physical and simulated seeding tests was significant, with the deviation increasing as the speed increased. This is due to the increased external disturbances at higher speeds, which caused larger deviations in the data. As the rotation speed increased from 5 r/min to 15 r/min, the mean seed-metering deviation in the physical test increased from 1.5010 mm to 1.9460 mm, with a similar trend observed in the simulated tests (from 1.3472 mm to 1.6617 mm). These findings indicate that rotational speed significantly impacts seed-metering deviation. Statistical tests showed no significant differences in data distribution (K-S test, p-value > 0.05) or mean deviation (Welch’s t-test, p-value > 0.05) between the two groups.

Based on the findings in Tables 16 and 17, it is evident that the performance of the physical seeding test evaluation indices is generally lower than that of the simulated seeding test, with higher data dispersion and greater coefficient of variation observed in the physical tests. The reasons for these discrepancies are as follows:

The multi-sphere aggregation model used in this study can approximate the overall shape of the seeds but cannot fully replicate the complex irregular geometry and surface texture of real spinach seeds. As a result, during the physical seeding test, the friction and collision behaviors in the seed-filling process differ from the simulation. Spinach seeds, due to their irregular shape, are more prone to mutual support in the filling zone. When accumulated seeds collapse, multiple seeds can simultaneously fill a single seed-metering hole, leading to a slightly higher multi-seed excess rate in the physical test compared to the simulation. Additionally, the irregular-shaped seeds experience uneven forces in the seed-metering holes, which may cause them to be prematurely scraped out by the seed-clearing brush or get stuck in the seed-filling zone, preventing proper discharge. This results in a higher single-seed deficit rate and empty hole rate in the physical test, causing the qualified seeding rate to be lower than in the simulation.

The core assumption of the spinach seed DEM models is that particles behave as rigid bodies and only undergo elastic collisions. However, real spinach seeds undergo plastic deformation during the seeding process, making their motion trajectory more random. Therefore, the stability of the simulated seeding test is better than that of the physical seeding test. In the simulation, all seed contact parameters are set as fixed values, but spinach seeds within a population differ in properties such as moisture content and surface smoothness, leading to significant variations in mechanical parameters. As a result, simulated seeding test indices are generally higher than those from the physical seeding test.

Micro-vibrations from the motor and transmission mechanism could cause seeds that are already placed in the seed-metering holes to vibrate out (increasing the single-seed deficit rate and empty hole rate) or lead to extra seeds being inadvertently shaken into the holes (increasing the multi-seed excess rate).

Manufacturing and assembly errors, such as variations in part surface finish and gaps with the housing, which are idealized in the simulation, directly affect the stability of seed flow. These factors result in simulated seeding test indices being higher than those from the physical seeding test. Spinach seeds are sensitive to moisture content.

When environmental humidity increases, the moisture content on the seed surface rises, which increases the friction coefficient between spinach seeds, leading to higher seed flow rate, empty hole rate, and multi-seed excess rate in the physical test. The increased friction between the seeds and the seed-metering device’s inner walls can also lead to increased seeding delays, resulting in a higher qualified seeding rate in the simulated seeding test than in the physical test.

In dry laboratory environments, the lightweight and small mass of spinach seeds make them prone to electrostatic charges due to friction, causing seeds to adhere to the walls of the seed-metering device. The DEM model does not include electrostatic attraction forces, which leads to smoother seed flow in the simulation than in reality. Additionally, temperature fluctuations could cause slight thermal expansion of the seed-metering device, alter the seed-metering hole dimensions and reduce the stability of the physical seeding test.

## Discussion

The integrated “calibration–simulation–optimization” workflow established in this study offers substantial value for industrializing spinach precision seeders. Using the discrete element method as a virtual prototyping tool significantly reduces the need for repeated physical prototyping and debugging, thereby shortening development cycles and lowering R&D costs. Sensitivity analysis further identified the seed-metering hole width (*D*_*h*_) as the most influential parameter on seeding qualified seeding rate—a finding that provides clear guidance for manufacturers to prioritize machining accuracy and wear resistance in production. Furthermore, the calibrated high-fidelity DEM model functions as a functional digital twin of the seed-metering device, laying a foundational model basis for future adaptive seeding systems that can recognize seed varieties and autonomously adjust operational parameters following a “one-variety-one-parameter” paradigm.

The core contribution of this work lies in its targeted response to seeding challenges specific to spinach—challenges that prevent the direct adoption of metering solutions designed for large-grained crops such as corn or wheat. These include the seed’s irregular shape and low sphericity, which increase the risk of clogging and overlapping during filling; its small size and low mass, which make the seed population susceptible to disturbances such as vibration or airflow; and the agronomic requirement of placing two seeds per hole, which demands higher precision in hole capacity and filling reliability than conventional single-seed metering. By developing a dedicated DEM parameter set and introducing an optimized rectangular seed hole tailored to dual-seed filling, this study effectively addresses these compounded challenges and fills a critical gap in specialized seeding models for small and irregularly shaped seeds.

While this study provides a robust framework, certain limitations warrant consideration. The DEM simulations are computationally demanding, and their resource-intensive nature can preclude extensive parametric studies or the analysis of highly complex scenarios. Furthermore, the multi-sphere approximation of seed geometry, though computationally efficient, constitutes a significant simplification that may not fully elucidate micro-scale mechanical interactions due to unmodeled surface features. Perhaps most critically, the performance validation conducted under controlled laboratory conditions has not yet been extended to the dynamic and unpredictable field environment, where factors such as soil heterogeneity and machine vibration could influence operational efficacy.

## Conclusions

The core innovation of this study lies in the tight integration of seed-metering device design with the discrete element method, addressing spinach-specific challenges—small particle size, irregular morphology, and the two-seeds-per-hole requirement—thereby significantly enhancing seeding accuracy and efficiency. First, we established a spinach-tailored DEM calibration workflow: by combining physical tests (angle of repose, mass flow rate) with iterative simulations, we precisely calibrated contact parameters for seed-seed and seed-material (PVC) interactions. This improves the accuracy of seed behavior simulations and provides quantitative data support for structural optimization, avoiding biases inherent in experience-driven design. Second, we developed a targeted structural innovation: an optimized “two-seeds-per-hole + rectangular seed-metering hole” scheme. It overcomes the single-seed capacity limitation of conventional holes by matching hole volume to seed dimensions, enabling each hole to reliably accommodate two seeds. Meanwhile, replacing circular holes—prone to jamming and overlap with small, irregular seeds—with rectangular holes, whose straight sidewalls better conform to spinach seed morphology, reduces mechanical interlocking during seed filling and stabilizes seed pickup and release.

Compared with the physical bench test results of the versatile vegetable seed-metering device, the optimized device demonstrates measurable improvements: at 10 r/min, the physical qualified seeding rate increases from 87.79% to 92.23% (a gain of approximately 4.44% points), the physical missed-seeding rate decreases from 9.20% to 6.33% (a reduction of approximately 2.87% points), and the physical multi-seed excess rate drops from 3.01% to 1.44% (a decrease of approximately 1.57% points). These enhancements collectively ensure improved seeding accuracy, operational stability, and inter-hole/intra-hole uniformity.

Based on these findings, we propose four engineering recommendations: (1) adopt the two-seeds-per-hole design strategy and dimension the seed-metering holes (depth, length, and width) according to the measured triaxial seed size to ensure stable double occupancy; (2) implement rectangular seed-metering holes to minimize jamming and overlapping; (3) use a seed-filling angle of 37.99° and a rotational speed of approximately 10 r/min as primary operational parameters to balance precision and efficiency; and (4) emphasize accurate DEM contact parameter calibration and prioritize the machining accuracy and wear resistance of the seed-metering hole width (*D*_*h*_), identified as the most sensitive dimensional parameter, to improve product consistency and durability.

For practical deployment and extension, future work will focus on three directions: field validation—mounting the optimized device on planters to systematically evaluate performance under real-world conditions (soil resistance, plot unevenness, and vibration coupling); model advancement—incorporating high-fidelity seed geometry via 3D scanning/CT and integrating environmental factors (e.g., moisture, electrostatics) into constitutive and contact models to enhance cross-scenario adaptability; and application expansion—applying the “calibration-simulation-optimization” framework to other high-value, small-seed vegetables (e.g., lettuce, rapeseed) to develop dedicated seed-metering components and parameter libraries, supporting “one variety, one parameter” adaptive precision seeding.

## Data Availability

The datasets generated during and/or analyzed during the current study are available from the corresponding author on reasonable request.

## References

[1] Vargas-Murga, L., De Rosso, V. V., Mercadante, A. Z. & Olmedilla-Alonso, B. A. Fruits and vegetables in the Brazilian household budget survey (2008–2009): carotenoid content and assessment of individual carotenoid intake. *J. Food Compos. Anal.* 88–96. (2016).

[2] Zheng, G. et al. Calibration and testing of discrete element simulation parameters for spinach seeds. *Comput. Part. Mech.* 1–12. (2024).

[3] Li, H., Lu, J., Cheng, B. & Song, W. Effects of film overlying soil technology on soil heat balance for mechanized vegetable planting: experimental study and numerical modelling. *Sol Energy*. **278**, 112786–112786 (2024).

[4] Liu, W. et al. Key technologies in intelligent seeding machinery for cereals: recent advances and future perspectives. *Agriculture* **15**, 8–8 (2024).

[5] Zhou, L. et al. Validation and Calibration of maize seed–soil inter-parameters based on the discrete element method. *Agronomy* **13**. (2023).

[6] Haikang, L. et al. Research status and future trends of DEM in seed metering process of seeders[J]. *Int. J. New. Dev. Eng. Soc. ***9**(1) (2025).

[7] Maraveas, C., Tsigkas, N. & Bartzanas, T. Agricultural processes simulation using discrete element method: a review[J]. *Comput. Electron. Agric. ***237**(PC), 110733–110733 (2025).

[8] Dongxu, Y. et al. A review of the application of discrete element method in agricultural engineering: A case study of Soybean[J]. *Processes* **10**(7), 1305–1305 (2022).

[9] Tian, Y. et al. Simulation of tensile behavior of tobacco leaf using the discrete element method (DEM). *Comput. Electron. Agric.* **205**. (2023).

[10] Wei, J. et al. Calibration and experimental validation of contact parameters in a discrete element model for tobacco strips. *Processes ***10**, 998–998 (2022).

[11] Zhang, S. et al. Discrete element modeling and shear properties of the maize stubble-soil complex. *Comput. Electron. Agric.* **204** (2023).

[12] Zheng, J., Wang, L., Wang, X., Shi, Y. & Yang, Z. Parameter calibration of cabbages (Brassica Oleracea L.) based on the discrete element method. *Agriculture* **13**, 555–555 (2023).

[13] Jinming, Z., Lin, W., Xiaochan, W., Yinyan, S. & Zhenyu, Y. Parameter calibration of cabbages (Brassica Oleracea L.) based on the discrete element method. *Agriculture* **13**, 555–555 (2023).

[14] Guopeng, M. et al. Measurement of physical properties of sorghum seeds and calibration of discrete element modeling parameters. *Agriculture* **12**, 681–681 (2022).

[15] Wang, X., Zhai, Q., Zhang, S., Li, Q. & Zhou, H. Efficient and accurate calibration of discrete element method parameters for black beans. *Agronomy* **14**, 2803–2803 (2024).

[16] Chen, Z., Xue, D., Guan, W., Guo, J. & Liu, Z. Performance Optimization of a spoon precision seed metering device based on a maize seed assembly model and discrete element method. *Processes* **11** (2023).

[17] Deng, T. et al. Analytical design and test of a clip-based precision corn seed metering device using DEM-MBD coupling. *Sci. Rep.* **15**, 9349 (2025). 40102633 10.1038/s41598-025-92833-9 PMC11920418

[18] Zheng, G. et al. Engineering discrete element Method-Based design and optimization of the key components of a Spoon-Wheel spinach Seed-Metering device. *Agronomy* **14**, 2096–2096 (2024).

[19] Yansong, S., Bo, Z., Jitao, Y. & Shun, Z. Design and experiment of impeller seed guide device for rice internal Suction hole direct seeding device. *Sci. Rep.* **14**, 13300–13300 (2024). 38858428 10.1038/s41598-024-64002-x PMC11639696

[20] Horváth, D., Poós, T. & Tamás, K. Modeling the movement of hulled millet in agitated drum dryer with discrete element method. *Comput. Electron. Agric.* **162**, 254–268 (2019).

[21] Bryan, A. J., Marolo, A., Chen, Y. & Zeng, Z. Calibration of discrete element parameters of crop residues and their interfaces with soil. *Comput. Electron. Agric.* **188**. (2021).

[22] Fan, G., Wang, S., Shi, W., Gong, Z. & Gao, M. Simulation Parameter calibration and test of typical pear varieties based on discrete element method. *Agronomy ***12**, 1720–1720 (2022).

[23] Jalal, K., Joanna, W. & Herman, R. Modelling and simulation of fruit drop tests by discrete element method. *Biosyst Eng.* **212**, 228–240 (2021).

[24] Zhong, J. et al. Determination and interpretation of parameters of double-bud sugarcane model based on discrete element. *Comput. Electron. Agric.* **203** (2022).

[25] Xu, C., Xu, F., Tang, H. & Wang, J. Determination of characteristics and establishment of discrete element model for whole rice plant. *Agronomy* **13 **(2023).

[26] Yan, D., Yu, J., Wang, Y., Zhou, L. & Tian, Y., & Zhang, N. Soil particle modeling and parameter calibration based on discrete element method Vol. 12, 1421–1421 (Agriculture, 2022).

[27] Wang, Q., Mao, H. & Li, Q. Modelling and simulation of the grain threshing process based on the discrete element method. *Comput. Electron. Agric.* **178**, 105790 (2020).

[28] Shen, C., Li, Y. & Xu, X. Calibration of Discrete Element Simulation Parameters for Powder Screw Conveying. J. Eng. 2021. (2021).

[29] Park, D., Lee, C. G., Park, H., Baek, S. H. & Rhee, J. Y. Discrete element method analysis of the impact forces on a Garlic bulb by the roller of a Garlic harvester. *J. Biosyst Eng.* **44**, 208–217 (2019).

[30] Park, D. et al. Analysis of Inter-particle contact parameters of Garlic cloves using discrete element method. *J. Biosyst Eng.* 1–14. (2021).

